# Preparation and Performance Evaluation of Temperature-Resistant and Salt-Resistant Zwitterionic Polymer Gel

**DOI:** 10.3390/gels12090819

**Published:** 2026-09-06

**Authors:** Meilong Fu, Shoufei Lu, Yangjie Fan, Yuxin Bai

**Affiliations:** College of Petroleum Engineering, Yangtze University, Wuhan 430100, China

**Keywords:** amphoteric polymer, gel, temperature and salt resistance, enhance oil recovery, performance evaluation

## Abstract

To address the susceptibility of polymer gels to syneresis and failure under high-temperature, high-salinity (HTHS) reservoir conditions, a zwitterionic terpolymer was synthesized from acrylamide (AM), 2-acrylamido-2-methylpropanesulfonic acid (AMPS), and dimethyldiallylammonium chloride (DMDAAC), and subsequently crosslinked with a phenolic system to produce an intramolecular salt-structured polymer gel with exceptional thermal and saline tolerance. Through systematic formulation optimization, the optimal composition was established as 0.3 wt% zwitterionic polymer, 0.03 wt% resorcinol, 0.3 wt% hexamethylenetetramine, and 0.1 wt% thiourea. X-ray photoelectron spectroscopy and zeta potential measurements confirmed that quaternary ammonium cations and sulfonate anions on the polymer chains associate via electrostatic attraction to form intramolecular salt bridges, which induce an anti-polyelectrolyte effect and thereby confer superior salt tolerance and thermal stability. Following aging for 140 days at 115 °C in formation water, the gel exhibited a syneresis rate below 30%, demonstrating favorable long-term stability under the tested high-temperature and high-salinity conditions. Thermogravimetric analysis and differential scanning calorimetry further characterized the thermal-transition behavior of the gel under programmed heating conditions. Scanning electron microscopy revealed a homogeneous honeycomb-like porous crosslinked network. Rheological testing demonstrated a storage modulus (G′) of 1.115 Pa and a loss modulus (G″) of 0.205 Pa, indicating an elasticity-dominated viscoelastic response and strong resistance to shear deformation. Single-core plugging experiments showed that at an injection volume of 0.2 PV, the gel achieved a plugging efficiency of 85.15% and a breakthrough pressure gradient of 18.6 MPa/m, with significant secondary plugging capability. In a heterogeneous dual-core model with a permeability contrast of approximately 10, the diversion rate into the low-permeability layer increased to 73.6%, effectively improving the water injection profile. This intramolecular salt-structured molecular design demonstrates potential for deep-profile control under high-temperature and high-salinity reservoir conditions.

## 1. Introduction

Excessive water production represents one of the most critical challenges encountered in mature oilfields worldwide. It not only compromises the economic efficiency of oil production but can also accelerate the abandonment of production wells [1,2,3,4,5]. A widely implemented strategy to mitigate this issue is profile control and water shutoff technology, which involves the injection of plugging agents into reservoirs to block high-permeability preferential flow pathways, divert injected fluids toward previously unswept zones, improve water sweep efficiency, and enhance the recovery of bypassed oil. Among various plugging materials, polymer gels have become the most extensively developed and applied chemical systems due to their low cost, operational simplicity, and excellent plugging performance [6,7,8,9,10]. Polymer gels are three-dimensional crosslinked elastomeric networks primarily composed of polymers and crosslinking agents, along with various functional additives. Their porous network structures can absorb and retain large quantities of water, enabling them to withstand considerable formation pressures and provide effective reservoir conformance control [11,12,13].

With the continuous depletion of recoverable reserves in conventional oilfields, increasing research attention has been devoted to reservoirs characterized by severe operating conditions, particularly high-temperature and high-salinity environments [14,15]. The Jianghan Oilfield serves as a representative example, where reservoir temperatures typically range from 115 to 150 °C and the total dissolved solids (TDS) concentration of formation water generally exceeds 2.5 × 10^4^ mg/L, with even higher salinity levels occurring in certain blocks. In the present study, experimental evaluation was conducted primarily at 115 °C, corresponding to the representative test temperature selected for the target reservoir conditions. These reservoirs are classified as ultra-high-temperature and high-salinity reservoirs, presenting substantial challenges for effective development. Under such extreme conditions, conventional polyacrylamide-based gels often suffer from insufficient thermal stability and are susceptible to hydrolysis, molecular degradation, and dehydration. Moreover, highly saline formation brine can disrupt gel network structures, while divalent ions (e.g., Ca^2+^ and Mg^2+^) may interact with polymer chains through complexation, causing polymer aggregation and deterioration of gel properties. Consequently, conventional polymer gels are often unable to satisfy the requirements for long-term reservoir conformance control and enhanced oil recovery under harsh reservoir conditions [16,17,18]. Therefore, the development of polymer gels with long-term stability under high-temperature and high-salinity conditions is of great significance for improving oil recovery efficiency.

Polymer gels applied in oil and gas reservoirs are continuously subjected to elevated temperatures and highly saline environments throughout their service life. Excessive temperature and salinity can disrupt polymer molecular structures, resulting in significant deterioration of gel stability, mechanical strength, and plugging performance. Therefore, maintaining the structural integrity and desirable mechanical properties of polymer gels under such extreme conditions remains a key scientific and engineering challenge [19,20,21,22,23]. To overcome these limitations, extensive efforts have been devoted to enhancing gel performance through three major strategies: modification of polymer molecular structures, optimization of crosslinking systems, and incorporation of functional additives. Modified polyacrylamides, including hydrophobically modified polyacrylamide (HMPAM), zwitterionic polyacrylamide, and copolymers based on acrylamide (AM) and 2-acrylamido-2-methylpropanesulfonic acid (AMPS), have demonstrated considerable improvements in thermal resistance, salt tolerance, and mechanical strength.

Although zwitterionic terpolymers comprising acrylamide (AM), 2-acrylamido-2-methylpropanesulfonic acid (AMPS), and dimethyldiallylammonium chloride (DMDAAC) have been extensively studied as rheology modifiers, fluid-loss additives, and thickeners for drilling and fracturing fluids [24,25,26], their utilization as in-situ gelation systems for deep-reservoir profile control remains largely unexplored. Existing phenolic crosslinked gel systems predominantly rely on partially hydrolyzed polyacrylamide (HPAM) or conventional AM/AMPS copolymers as the polymer backbone, neither of which possesses an intrinsic zwitterionic intramolecular salt structure. As a result, these systems are prone to charge shielding and syneresis under high-salinity conditions, and they exhibit inadequate long-term thermal stability at temperatures above 115 °C. Moreover, prior investigations of AM/AMPS/DMDAAC polymers have focused primarily on solution-phase properties—such as viscosity retention and fluid-loss control—while largely neglecting the synergistic interplay between zwitterionic intramolecular salt bonds and covalent crosslinked networks in modulating gel viscoelasticity, shear recovery behavior, and secondary plugging performance. To address these knowledge gaps, this study proposes a differentiated strategy: integrating the AM/AMPS/DMDAAC zwitterionic terpolymer with a low-toxicity phenolic crosslinking system and a thiourea-based thermal stabilizer.

In this study, a temperature- and salt-resistant zwitterionic polymer gel was developed for application in ultra-high-temperature and high-salinity reservoirs. Based on a conventional AM/AMPS copolymer system, dimethyldiallylammonium chloride (DMDAAC) was introduced to synthesize an AM/AMPS/DMDAAC zwitterionic terpolymer. By combining this polymer with a phenolic crosslinking system and a thermal stabilizer, a high-performance zwitterionic gel was successfully prepared. The electrostatic interactions between quaternary ammonium and sulfonate groups establish an intramolecular salt structure that fundamentally distinguishes this gel from conventional phenol-crosslinked polyacrylamide and AM/AMPS copolymer systems. Unlike single-network gels that rely exclusively on covalent crosslinking, these intramolecular salt bonds function as reversible, sacrificial physical crosslinks capable of synergizing with the phenolic covalent network to enhance the thermal stability of the three-dimensional architecture. Under high-temperature, high-salinity conditions, external salt ions screen the electrostatic interactions within the intramolecular ion pairs; however, the anti-polyelectrolyte effect drives polymer chain extension—a phenomenon that has rarely been leveraged in gel-based profile control applications. This chain expansion effectively counteracts charge shielding and suppresses gel syneresis, thereby conferring exceptional salt tolerance upon the material. Furthermore, the dynamic reversibility of intramolecular salt bonds enables energy dissipation through bond breaking and reformation under shear deformation, endowing the gel with remarkable shear resistance and secondary plugging performance.

Comparative analysis against existing high-temperature, high-salinity (HTHS) gel systems further validates the competitive advantage of the formulation developed in this study. Li et al. [27] reported an AM/AMPS/PEI gel reinforced with 0.5 wt% nylon fiber that maintained a syneresis rate below 2.5% after 120 days at 130 °C and 240,720 mg/L salinity; however, this performance required a polymer concentration of 1.0 wt%. By contrast, the present zwitterionic terpolymer gel achieves effective plugging at merely 0.3 wt% polymer—a 50–70% reduction in dosage—while maintaining a syneresis rate below 30% after 140 days at 115 °C in actual formation water (total salinity 2.486 × 10^5^ mg/L). Moreover, its breakthrough pressure gradient of 18.6 MPa/m substantially exceeds values reported for conventional phenolic-crosslinked systems. For instance, HPAM crosslinked with water-soluble phenol-formaldehyde resin exhibited severe syneresis within 30 days at 140 °C, with gel strength degrading to Grade D [28]. Similarly, Guo et al. [29] demonstrated that high-strength PAtBA-PEI gels, despite exhibiting adequate stability at 130 °C in brine with salinities below 43,530 mg/L, underwent syneresis within 15 days at 110 °C once salinity exceeded this threshold. The superior performance of the present formulation stems from the intramolecular salt structure of the zwitterionic polymer, which synergizes with the phenolic covalent network to reinforce structural integrity under coupled thermal and chemical stress.

The thermal stability, microstructural characteristics, viscoelastic properties, and plugging performance of the developed gel were systematically investigated. The results demonstrate favorable performance of the proposed gel under the investigated high-temperature and high-salinity conditions at 115 °C, highlighting its potential for reservoir conformance-control applications under comparable conditions.

## 2. Results and Discussion

### 2.1. Optimization of Formulation of Gel System

#### 2.1.1. Concentration Optimization of Zwitterionic Polymer

**Figure 1:**
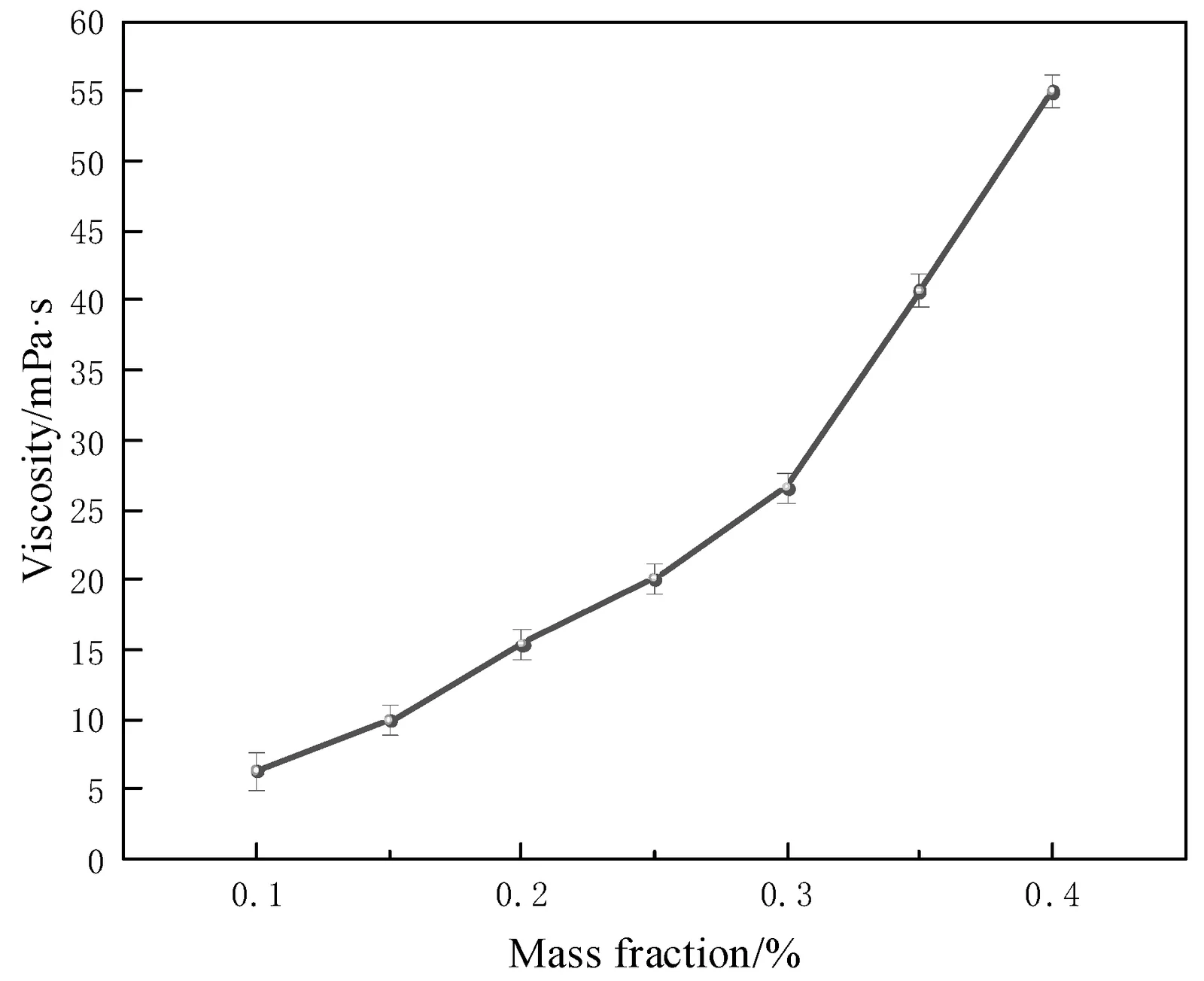
Viscosity-concentration diagram of zwitterionic polymer (115 °C).

The medium-porosity, low-permeability, and high-temperature/high-salinity reservoir conditions of the Jianghan Oilfield impose stringent requirements on the selection of polymer type and concentration for gel formulation. To develop a gel system suitable for field application, zwitterionic polymer solutions were prepared using formation water collected from the target reservoir. The optimal polymer concentration was determined based on the viscosity–concentration relationship of the polymer solutions. All measurements were performed in triplicate (n = 3), and data are presented as mean ± standard deviation. The experimental results are presented in Figure 1.

The viscosity–concentration relationship of the zwitterionic polymer solution is illustrated in Figure 1. When the polymer concentration exceeds 0.3 wt%, the solution viscosity increases sharply, resulting in substantially higher injection resistance and potentially compromising deep reservoir injectivity. In contrast, polymer concentrations below 0.3 wt% lead to insufficient solution viscosity, which limits gel strength after crosslinking and reduces the effectiveness of blocking high-permeability channels. Therefore, considering reservoir injectivity, plugging performance, and economic feasibility, a polymer concentration of 0.3 wt% was selected as the optimal formulation.

A sharp increase in viscosity was observed once the polymer mass fraction exceeded 0.3%. Below this threshold, individual polymer chains exist as isolated, non-interacting random coils within the dilute solution regime, yielding low solution viscosity. Under these conditions, insufficient chain entanglement occurs during crosslinking, preventing the formation of a robust gel network. At and above 0.3%, the random coils begin to overlap and interpenetrate, and the system transitions into the semi-dilute solution regime where chain entanglement is markedly enhanced. This entanglement-dominated state manifests macroscopically as a substantial viscosity increase, which ensures adequate gel strength upon crosslinking but simultaneously elevates injection resistance. Consequently, the 0.3% mass fraction represents the critical transition point from the dilute to the semi-dilute regime, striking an optimal balance between the inherently competing demands of injectability and gel strength.

#### 2.1.2. Concentration Optimization Experiment of Phenolic Cross-Linked Gel System

In conventional phenolic crosslinking systems, phenol-based crosslinkers may pose greater environmental and reservoir-related concerns due to their toxicity. Therefore, a low-toxicity crosslinking system consisting of hexamethylenetetramine (HMTA) and resorcinol was selected as an alternative. The crosslinking reaction between HMTA and resorcinol generates covalent bonds among polymer chains, thereby significantly improving the thermal stability of the gel network. Furthermore, the compact three-dimensional polymer network formed through covalent crosslinking enhances gel structural integrity, extends gel lifetime, and effectively suppresses dehydration (syneresis).

**Figure 2:**
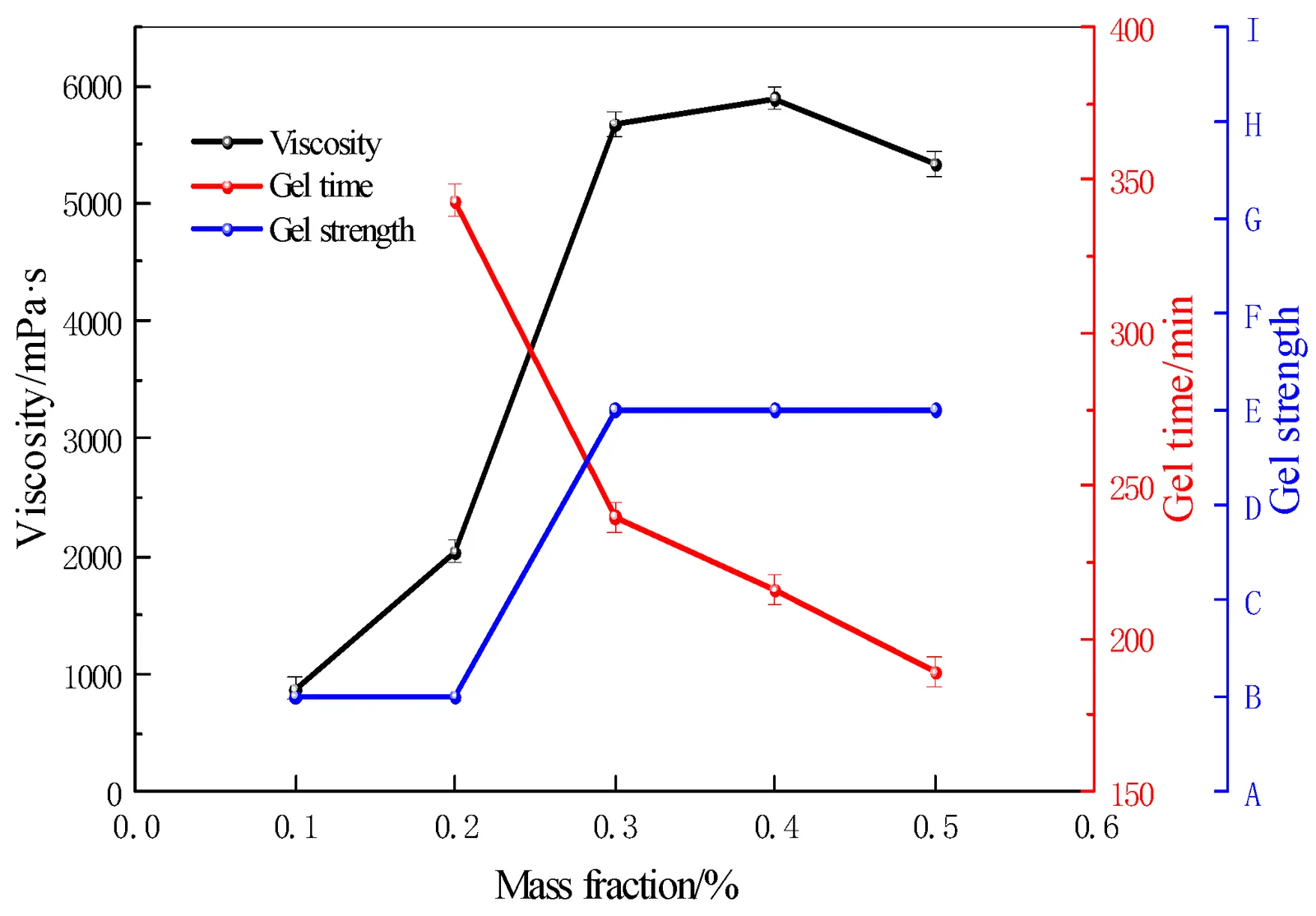
Effect of HMTA mass concentration on gel test results (115 °C).

Using the optimized 0.3 wt% zwitterionic polymer concentration as the base formulation, the concentrations of resorcinol and the high-temperature stabilizer thiourea were maintained at 0.03 wt% and 0.1 wt%, respectively. The HMTA concentration was varied from 0.1 wt% to 0.5 wt% across five levels to investigate its influence on gelation behavior at 115 °C. The optimal HMTA concentration was subsequently determined based on the gelation performance. All tests were carried out with three independent replicates (n = 3), and data are expressed as mean ± standard deviation. The experimental results are presented in Figure 2.

The experimental results demonstrate that HMTA concentration exerts a significant influence on the gelation time, gel strength, and viscosity of the gel system. As the HMTA concentration increased from 0.1 wt% to 0.3 wt%, the crosslinking reaction among polymer chains was gradually enhanced, resulting in the formation of a more interconnected gel network. Consequently, both gel viscosity and gel strength increased significantly, while the gelation time was correspondingly shortened.

When the HMTA concentration was increased from 0.3 wt% to 0.4 wt%, the gel viscosity reached its maximum value, whereas the gel strength remained relatively stable. Meanwhile, the gelation time was maintained within an appropriate range for field application, providing a balance between injectivity and effective in situ gel formation. However, a further increase in HMTA concentration to 0.5 wt% caused a substantial decrease in gel viscosity and strength, together with an excessively short gelation time. This phenomenon may be attributed to overly rapid crosslinking, which can result in an uneven gel network and reduced mechanical integrity. Considering the overall gel performance and operational requirements, an HMTA concentration of 0.3 wt% was selected as the optimal value.

**Figure 3:**
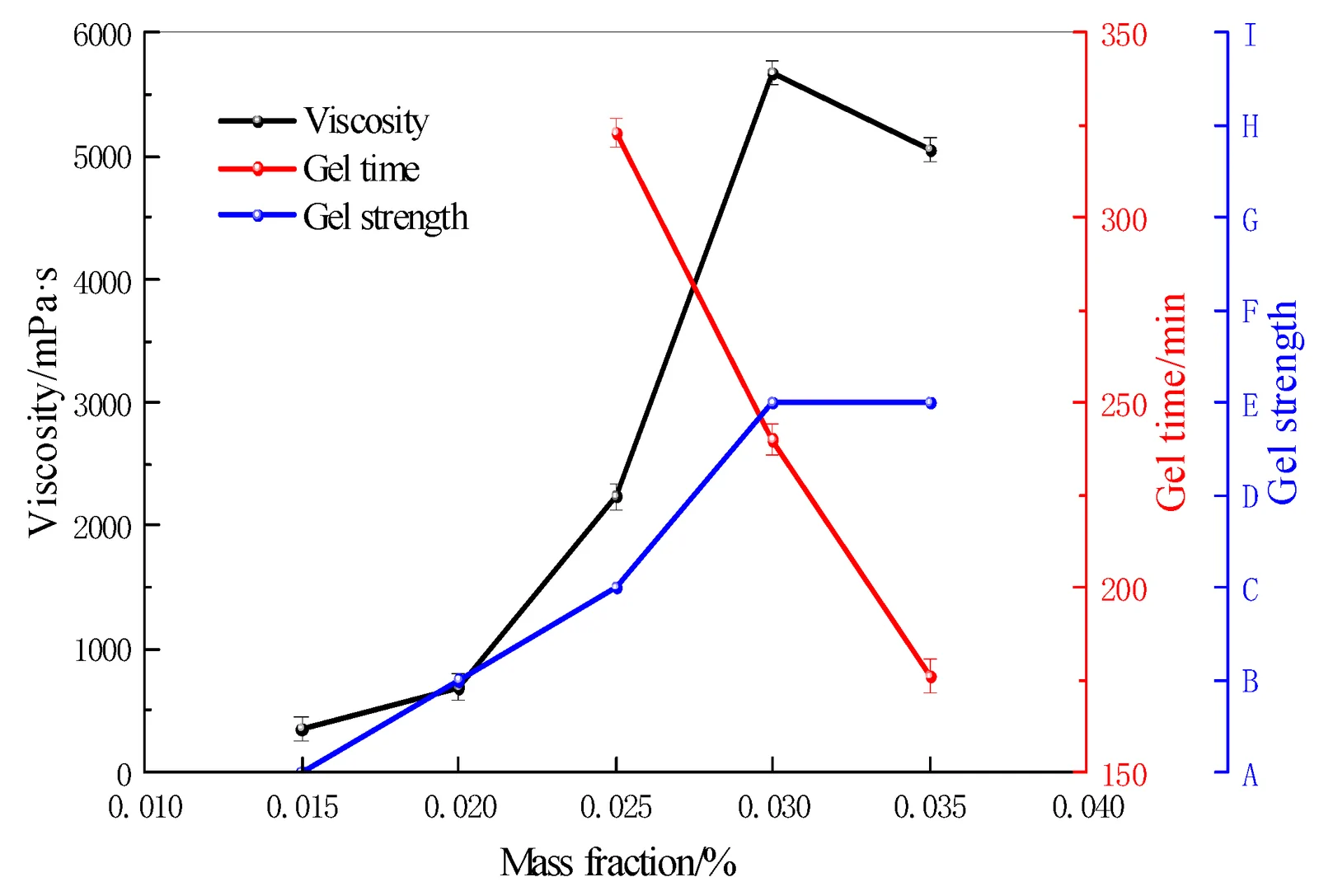
Effect of resorcinol mass concentration on gel test results (115 °C).

To further optimize the formulation of the phenolic crosslinked gel system, the optimal resorcinol concentration was investigated. The polymer concentration, HMTA concentration, and thiourea content were fixed at 0.3 wt%, 0.3 wt%, and 0.1 wt%, respectively, with thiourea serving as a high-temperature stabilizer. The resorcinol concentration was varied from 0.015 wt% to 0.035 wt% across five levels to evaluate its effects on gel strength and gelation time at 115 °C. The optimal resorcinol concentration was determined based on the experimental results, as shown in Figure 3. All tests were carried out with three independent replicates (n = 3), and data are expressed as mean ± standard deviation.

The results demonstrate that resorcinol concentration has a significant influence on the gelation time, gel strength, and viscosity of the system at 115 °C. As the resorcinol concentration increased from 0.015 wt% to 0.03 wt%, the crosslinking reaction was gradually accelerated, promoting the formation of a more compact and interconnected polymer network. Consequently, both gel viscosity and gel strength increased significantly, accompanied by a gradual reduction in gelation time.

At a resorcinol concentration of 0.03 wt%, the gel viscosity reached its maximum value, while the gel strength remained relatively stable. Meanwhile, the gelation time was maintained within an appropriate range for field applications, achieving a balance between favorable injectivity and effective in situ gelation for deep-profile control. However, further increasing the resorcinol concentration to 0.035 wt% resulted in an excessively rapid gelation process, which may hinder sufficient gel propagation into deep reservoir zones and reduce the effectiveness of profile control.

Considering gel performance, reservoir injectivity, and the requirements of deep-profile control applications, a resorcinol concentration of 0.03 wt% was selected as the optimal formulation.

#### 2.1.3. Optimization Experiment of High Temperature Stabilizer Concentration and Long-Term Stability Evaluation

Considering the medium-to-high-temperature and high-salinity reservoir conditions of the Jianghan Oilfield, the concentration of thiourea, used as a high-temperature stabilizer, plays a crucial role in determining the long-term thermal stability and anti-aging performance of the gel system. Therefore, a series of optimization experiments were conducted to identify the optimal thiourea concentration.

**Figure 4:**
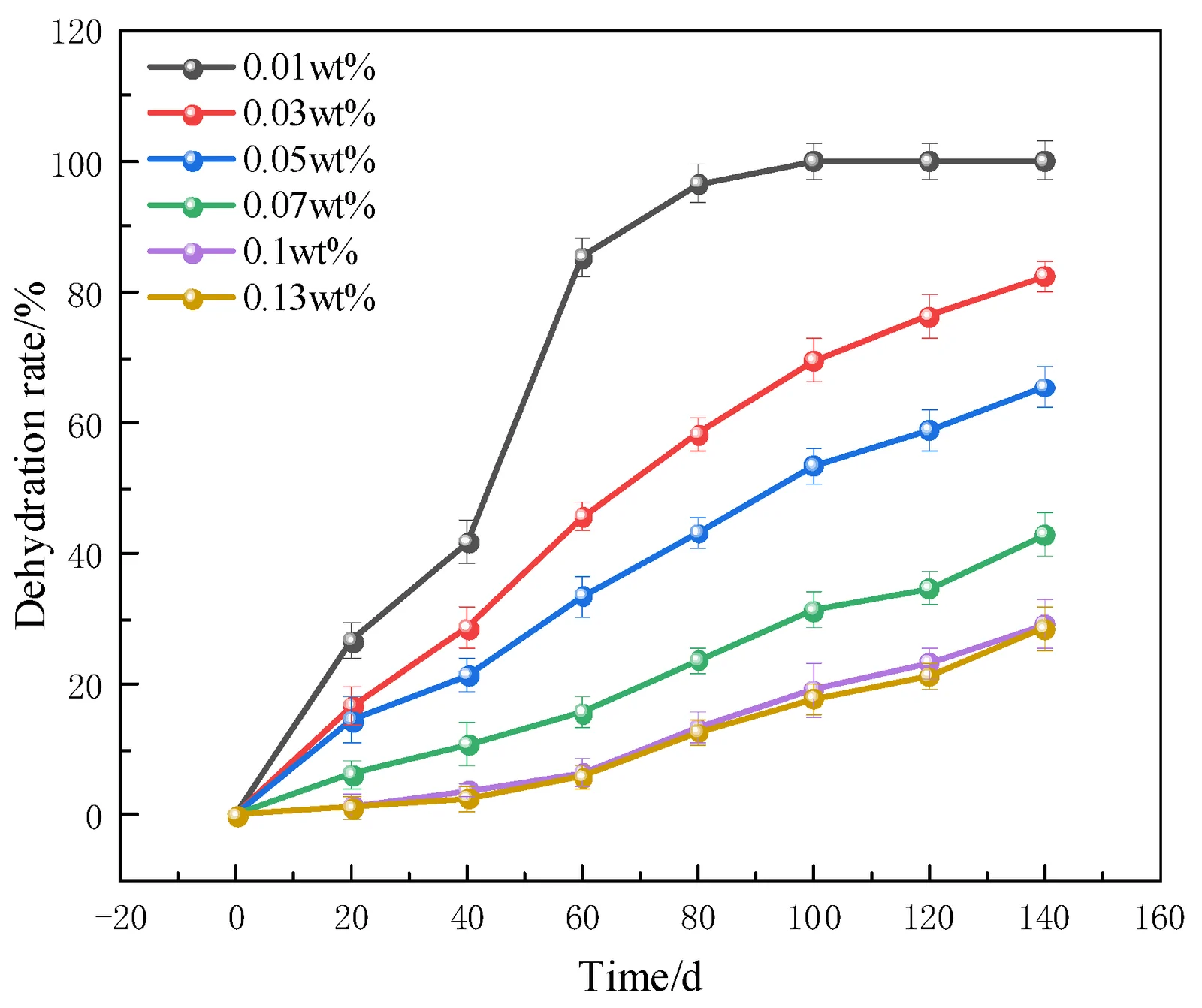
The test results of the effect of thiourea mass concentration on gel dehydration rate (115 °C).

The polymer concentration was fixed at 0.3 wt%, while the HMTA and resorcinol concentrations were maintained at 0.3 wt% and 0.03 wt%, respectively. The thiourea concentration was varied from 0.01 wt% to 0.13 wt% across multiple levels. Aging experiments were conducted at 115 °C to investigate the effect of thiourea concentration on the thermal stability of the gel system. The optimal thiourea concentration was determined based on the experimental results, as shown in Figure 4. All tests were carried out with three independent replicates (n = 3), and data are expressed as mean ± standard deviation.

The experimental results demonstrate that thiourea concentration significantly influences both the dehydration behavior and long-term thermal stability of the gel system. The dehydration rate of the thiourea-containing gels gradually increased with aging time; however, increasing the thiourea concentration effectively retarded the dehydration process and enhanced the long-term thermal stability of the gel under high-temperature conditions.

At a thiourea concentration of 0.01 wt%, the gel underwent complete dehydration after approximately 100 days of aging, indicating insufficient thermal stabilization for long-term profile control applications in the target reservoir. In contrast, when the thiourea concentration exceeded 0.1 wt%, the dehydration rate remained below 30% even after 140 days of aging. Furthermore, no significant differences in dehydration behavior were observed among formulations within this concentration range, suggesting that thiourea had reached an effective concentration threshold for providing high-temperature stabilization.

These results indicate that thiourea effectively suppresses the oxidative degradation of polymer chains and protects the integrity of the gel crosslinked network under high-temperature and high-salinity conditions. Consequently, gel dehydration and network shrinkage were significantly delayed. Considering both the long-term thermal stability of the gel system and the economic feasibility of field implementation, a thiourea concentration of 0.1 wt% was selected as the optimal formulation.

Long-term stability tests of the optimized gel formulation further confirmed that the selected thiourea concentration of 0.1 wt% enabled the gel system to maintain a dehydration rate below 30% after 140 days of aging at 115 °C. These results demonstrate favorable long-term stability under the investigated conditions of 115 °C and high salinity.

#### 2.1.4. Optimization Results of Gel System Formula

**Figure 5:**
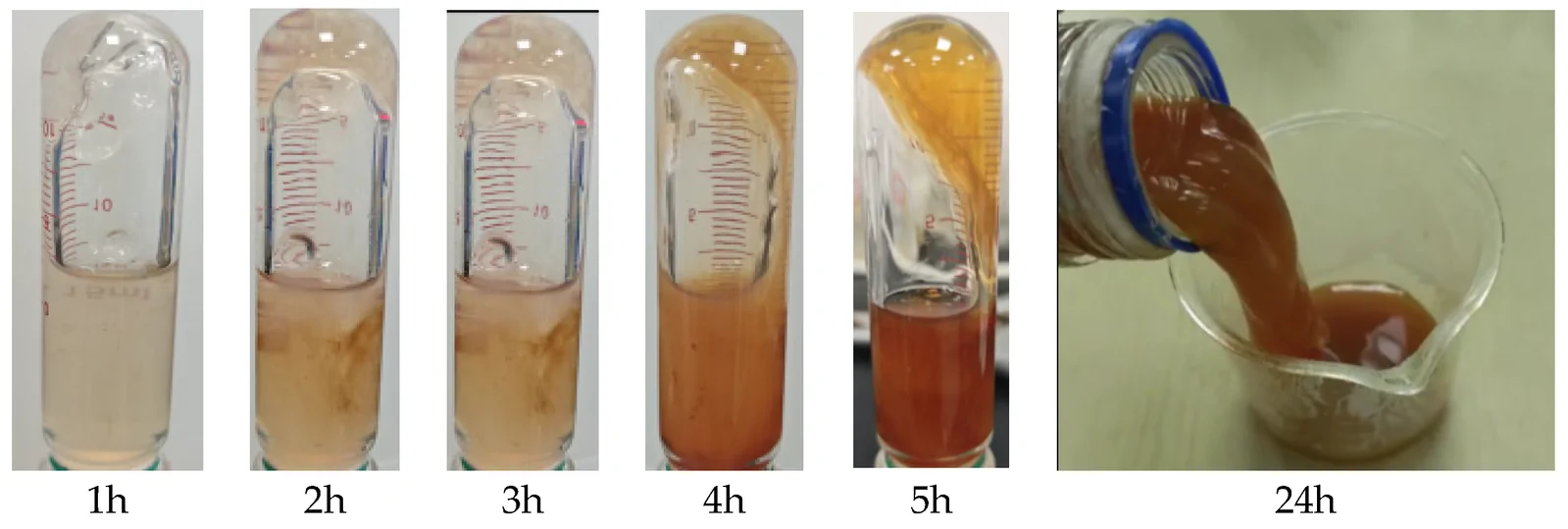
Gelling effect diagram of zwitterionic polymer gel (115 °C).

Finally, the optimal formulation of the zwitterionic polymer gel system for high-temperature and high-salinity reservoir applications was determined to consist of 0.3 wt% zwitterionic polymer, 0.03 wt% resorcinol, 0.3 wt% HMTA, and 0.1 wt% thiourea. Under reservoir conditions of 115 °C and high salinity, this formulation exhibited stable viscosity, appropriate gel strength, and excellent anti-aging performance (Figure 5), demonstrating its strong potential for application in the target reservoir of the Jianghan Oilfield.

### 2.2. Analysis of the Experimental Results of Thermogravimetric Analysis

**Figure 6:**
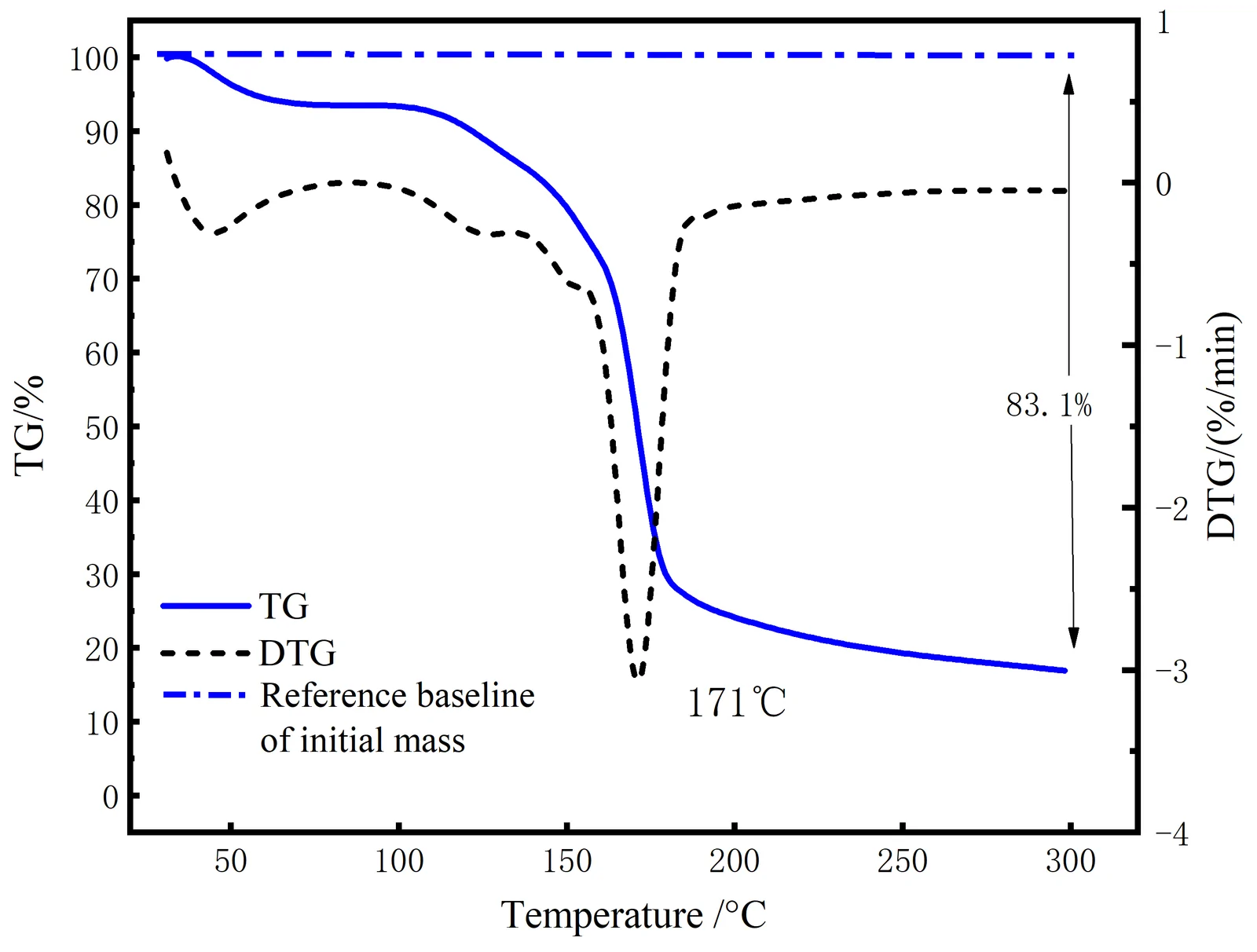
Thermogravimetric test curve of gel system.

The target reservoir in the Jianghan Oilfield exhibits medium-to-high temperature conditions with an average temperature of approximately 115 °C. To evaluate the thermal resistance and dehydration behavior of the gel system under programmed heating conditions, thermogravimetric analysis (TGA) was performed. TGA measurement was repeated twice independently, and the curve shown is a representative result with good reproducibility. The experimental results are presented in Figure 6.

The TG and DTG curves reveal three distinct stages of weight loss during the heating process. In the first stage, ranging from room temperature to 100 °C, the TG curve shows an extremely low weight loss rate, with the mass retention remaining above 95%. This stage is primarily associated with the evaporation of free water and physically adsorbed water within the gel. No significant chemical degradation occurs during this temperature range, and the crosslinked network structure remains intact, allowing the gel to maintain its water-rich three-dimensional network structure.

In the second stage, between 100 and 171 °C, a noticeable decrease in the TG curve is observed, accompanied by a maximum weight loss rate at approximately 171 °C in the DTG curve. This major weight loss stage is mainly attributed to the removal of bound water within the gel network. Meanwhile, partial cleavage of weak side-chain bonds may occur. However, the primary covalently crosslinked network remains stable without irreversible structural destruction within this temperature range. Therefore, 171 °C corresponds to the temperature at which the maximum dehydration rate is achieved.

When the temperature exceeds 200 °C, the decreasing rate of the TG curve becomes significantly slower, and the final mass retention stabilizes at approximately 20%. The remaining mass corresponds to the dried polymer matrix. At this stage, partial degradation of side groups begins, accompanied by initial deterioration of the crosslinked network; however, severe thermal decomposition of the polymer backbone does not occur within the investigated temperature range.

These results characterize the thermal weight-loss behavior of the developed gel under programmed heating conditions. The major mass-loss region below approximately 171 °C is mainly associated with water removal, while more pronounced degradation of the polymeric structure occurs at higher temperatures. However, these TGA results represent short-term thermal behavior under programmed heating and should not be interpreted as evidence of long-term hydrothermal stability at temperatures approaching 171 °C.

### 2.3. Differential Scanning Calorimetry (DSC) Analysis of Experimental Results

**Figure 7:**
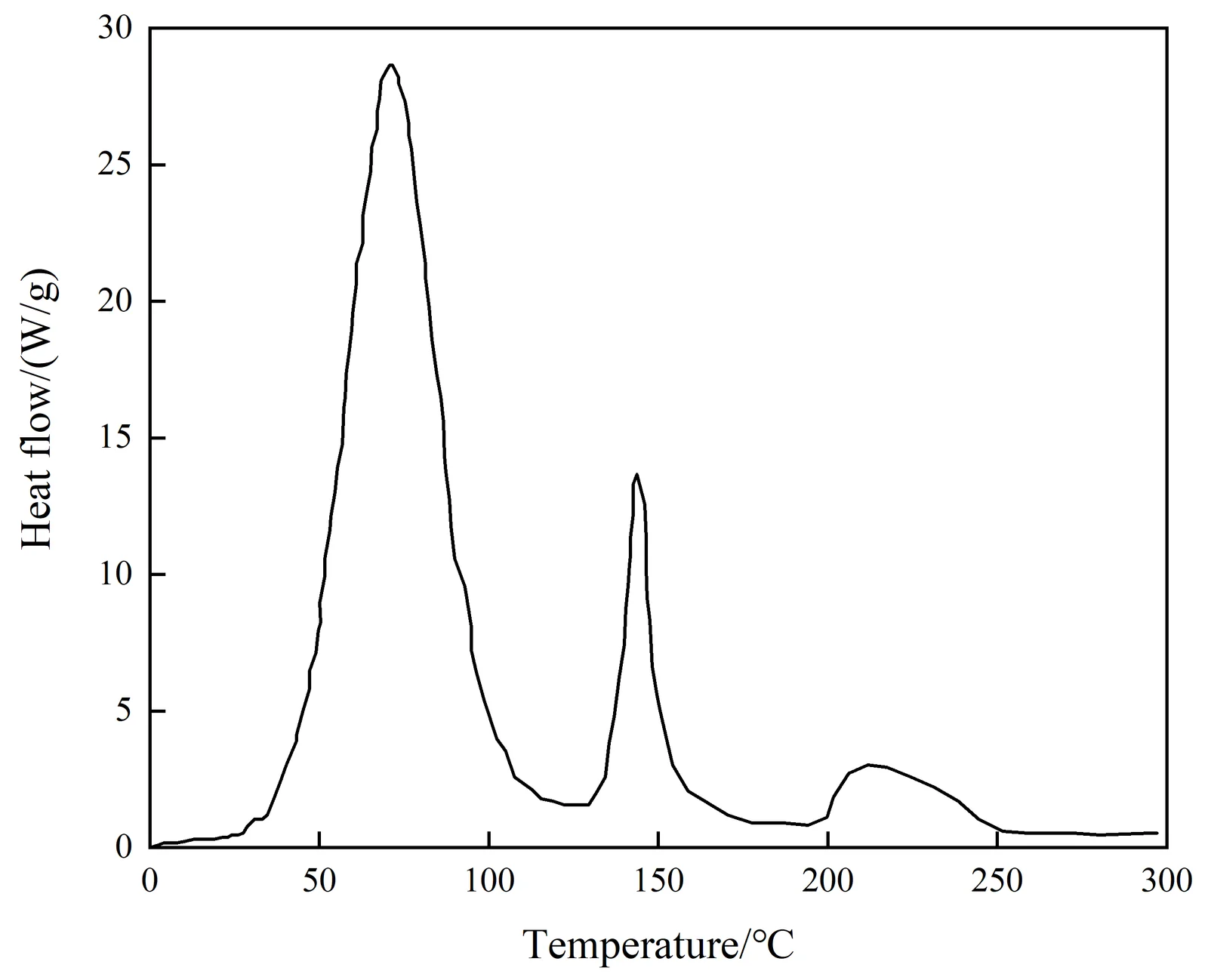
Differential scanning calorimetry (DSC) curve of the gel system.

DSC measurement was repeated twice independently, and the curve shown is a representative result with good reproducibility. Differential scanning calorimetry (DSC) analysis was performed to further investigate the thermal transition behavior of the gel system, and the results are presented in Figure 7. Within the investigated temperature range, the DSC curve exhibited three characteristic endothermic regions, and the thermal transition behavior showed good agreement with the thermogravimetric analysis (TGA) results.

The first pronounced endothermic peak appeared in the temperature range of 50–100 °C, with a maximum at approximately 70 °C. This peak was mainly attributed to the evaporation and removal of surface-adsorbed water and weakly bound free water within the gel network, representing a physical dehydration process. This observation is consistent with the TGA results, where only minor mass loss occurred below 100 °C and the mass retention remained above 95%. These results indicate that no significant chemical degradation occurred in this temperature range and that the crosslinked gel network maintained its structural integrity.

A second distinct endothermic peak was observed between 120 and 170 °C, with a peak temperature of approximately 145 °C. This thermal transition was primarily associated with the removal of bound water confined within the three-dimensional gel network, accompanied by the possible cleavage of weak bonds in polymer side chains. This temperature range corresponds well with the major mass-loss region observed in the TGA curve, further confirming that the maximum dehydration rate occurred near 171 °C. During this stage, the thermal response was dominated by the evaporation of physically and chemically bound water, while the primary covalently crosslinked network remained stable without irreversible thermal destruction.

A third broad and relatively weak endothermic peak appeared in the range of 200–250 °C, with a maximum at approximately 210 °C. This peak was associated with the decomposition of polymer side groups and the initial degradation of the crosslinked network. The relatively weak thermal effect indicates that the extent of degradation remained limited in this temperature range, which agrees well with the TGA results showing a significantly reduced mass-loss rate above 200 °C and a final mass retention of approximately 20%. These results suggest that the primary crosslinked framework possesses excellent thermal resistance. Overall, the DSC and TGA results characterize the thermal-transition behavior of the zwitterionic polymer gel under programmed heating conditions. Long-term thermal stability under saline conditions was experimentally demonstrated at 115 °C.

### 2.4. SEM Analysis of Experimental Results

**Figure 8:**
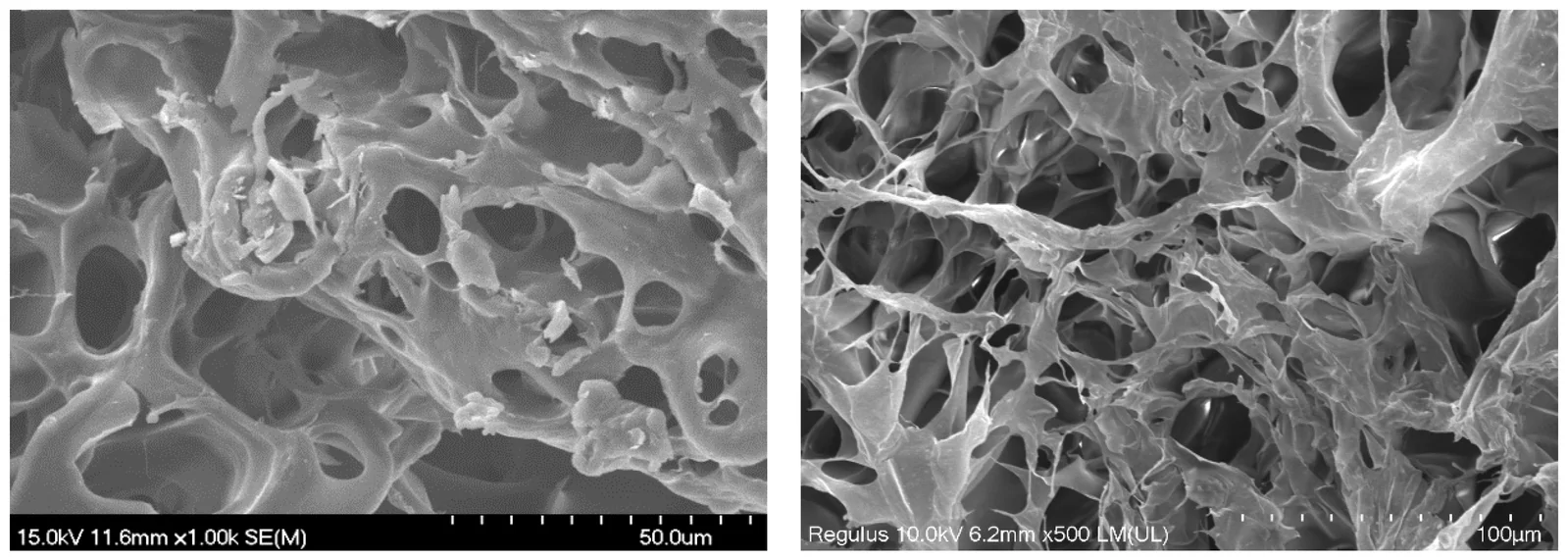
SEM morphology of zwitterionic polymer gel.

The microstructure of the gel after aging under high-temperature and high-salinity conditions (115 °C) was characterized using scanning electron microscopy (SEM), and the corresponding results are presented in Figure 8.

The SEM images reveal that the zwitterionic polymer gel possesses a relatively uniform honeycomb-like porous crosslinked structure with evenly distributed pores, compact and continuous pore walls, and no apparent cracks, fractures, or structural collapse. Furthermore, good interconnectivity among the pores can be observed throughout the gel matrix. The locally observed wrinkle-like and fused features suggest the formation of a highly interconnected polymer network, which may be attributed to the effective crosslinking interactions between polymer chains and the phenolic crosslinking system.

This stable three-dimensional network structure provides microscopic evidence supporting the excellent thermal stability observed in the previous TGA and contributes to the favorable injection and long-term plugging performance of the gel system. The uniform porous morphology helps maintain suitable permeability and avoid excessive injection resistance, while the compact pore walls and robust crosslinked network enhance resistance against deformation and erosion under high-temperature and high-salinity reservoir conditions.

Therefore, the well-preserved microstructure provides a structural foundation for the macroscopic viscoelastic properties and reservoir plugging performance of the gel system, demonstrating its potential applicability for medium-porosity and low-permeability reservoirs in the Jianghan Oilfield.

### 2.5. Analysis of XPS Test Results

**Figure 9:**
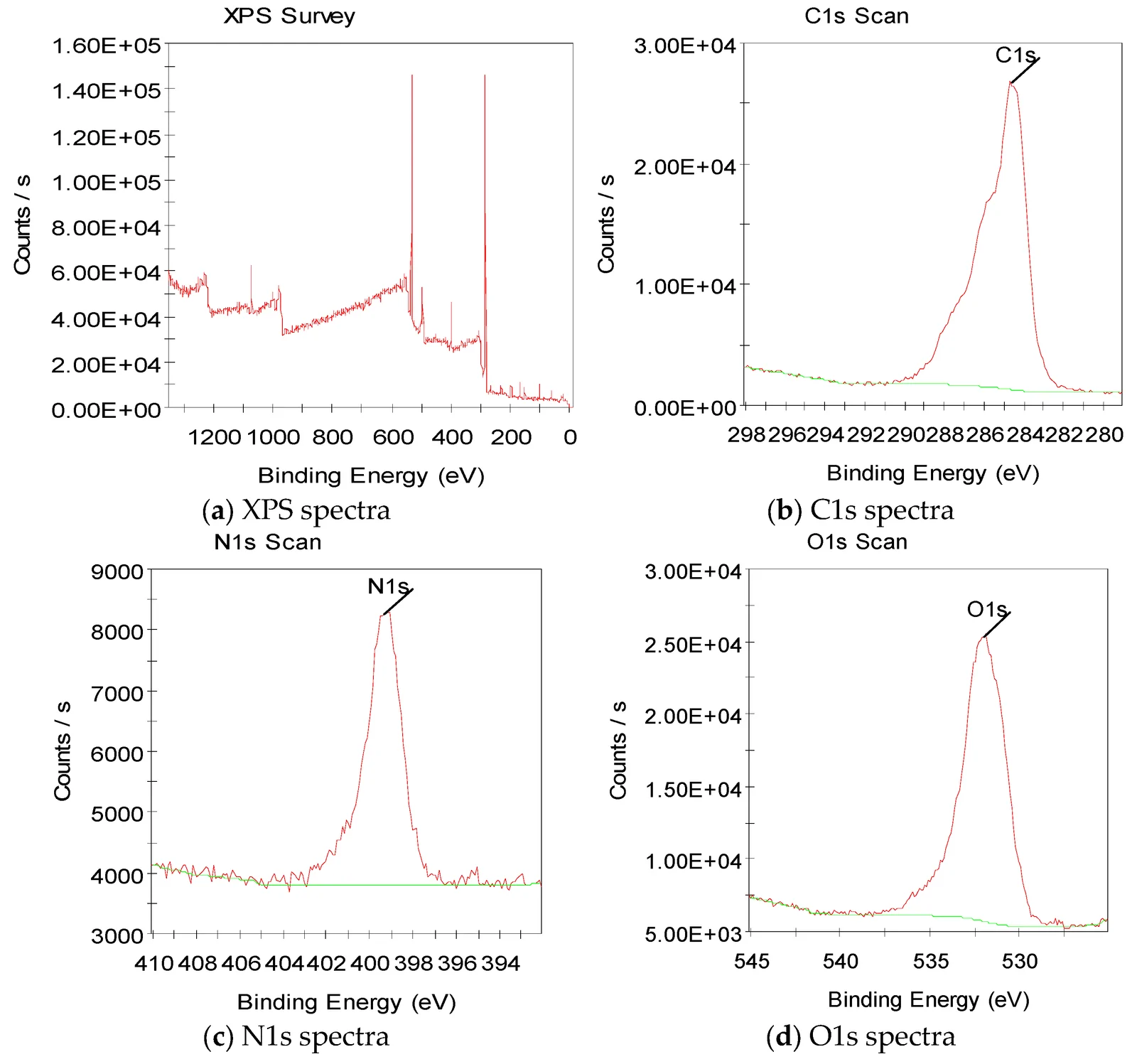
XPS test spectra of zwitterionic polymer gel.

X-ray photoelectron spectroscopy (XPS) was employed to investigate the surface chemical composition and bonding configurations of the zwitterionic terpolymer. The survey spectrum (Figure 9a) displays distinct photoelectron signals corresponding to C, N, O, and trace amounts of Si. Quantitative elemental analysis reveals atomic concentrations of 82.96% C, 3.18% N, and 13.38% O. The simultaneous presence of nitrogen- and oxygen-containing species provides evidence for the successful incorporation of AM, AMPS, and DMDAAC monomers into the polymer backbone.

The high-resolution C 1s spectrum (Figure 9b) was deconvoluted into three characteristic components. The dominant peak located at 284.36 eV is assigned to C–C and C–H bonds within the saturated aliphatic polymer backbone. The component at 286.02 eV is attributed to C–O and C–N bonds, corresponding to oxygen- and nitrogen-containing functional groups such as hydroxyl/ether and amide/amine moieties. The weak peak at 288.77 eV is associated with carbonyl carbon (C=O) species in amide groups.

The high-resolution N 1s spectrum (Figure 9c) exhibits an asymmetric peak envelope centered at approximately 400.8 eV, which can be fitted into two major components. The peak at 398.89 eV is assigned to neutral amide nitrogen (–CONH– and –CONH_2_) originating from AM units. In comparison, the dominant peak at 401.81 eV corresponds to quaternary ammonium nitrogen (–N^+^(CH_3_)_3_) derived from DMDAAC units. Notably, the binding energy of quaternary ammonium nitrogen (401.81 eV) is slightly lower than that typically reported for free quaternary ammonium salts (approximately 402–403 eV). This shift suggests that the positive charges are partially influenced by electrostatic interactions with neighboring anionic groups, providing spectroscopic evidence for the formation of an internal-salt structure. The high proportion of quaternary nitrogen further confirms the effective incorporation of cationic DMDAAC units into the polymer chain.

The high-resolution O 1s spectrum (Figure 9d) was fitted with two components. The dominant peak at 531.07 eV is attributed to oxygen species from sulfonate groups (–SO_3_^−^) in AMPS units and carbonyl oxygen (C=O) in amide groups. The shoulder peak at 532.50 eV corresponds to C–O–H and C–O–C species. The presence of sulfonate-related oxygen signals confirms the incorporation of AMPS units, whose negatively charged sulfonate groups serve as electrostatic counterparts to the quaternary ammonium groups. Furthermore, the slight shift in the O 1s binding energy compared with free sulfonate species indicates the existence of a modified electronic environment associated with internal ionic interactions.

Overall, the XPS results confirm that the zwitterionic terpolymer contains both positively charged quaternary ammonium groups and negatively charged sulfonate groups within the polymer chain. The coexistence of these oppositely charged functional groups and their characteristic binding-energy shifts provide strong evidence for the formation of internal ionic associations (–N^+^···^−^O_3_S–) through electrostatic interactions. This unique zwitterionic molecular architecture is considered responsible for the anti-polyelectrolyte effect of the gel system and provides the molecular basis for maintaining network integrity and salt tolerance under high-temperature and high-salinity reservoir conditions [30,31].

An obvious N 1s signal located near 400 eV confirms the presence of nitrogen-containing functional groups within the gel system. The nitrogen species mainly originate from amide groups (–CONH–/–CONH_2_) and quaternary ammonium groups introduced by the polymer components. Under high-temperature and high-salinity reservoir conditions, these nitrogen-containing groups can participate in intermolecular interactions, including ionic associations and hydrogen bonding, thereby contributing to the structural stability of the gel network and enhancing its salt tolerance.

In addition, the prominent O 1s signal near 530 eV indicates the presence of oxygen-containing functional groups, including hydroxyl (–OH), carbonyl (C=O), and ether (–O–) groups. These oxygen-containing moieties can interact with dissolved ions in formation brine. For monovalent cations such as Na^+^, the electronegative oxygen atoms can induce ion–dipole interactions through electrostatic attraction. For divalent cations such as Ca^2+^ and Mg^2+^, the lone-pair electrons of oxygen atoms may participate in coordination interactions with metal ions, forming additional physical association sites within the polymer network. These ionic interactions contribute to the stabilization of the gel structure under high-temperature and high-salinity conditions. Furthermore, hydrogen bonding among oxygen- and nitrogen-containing functional groups can effectively restrict polymer chain mobility, reduce network deformation, and improve the thermal stability of the gel system. The synergistic effects of ionic interactions, coordination interactions, and hydrogen bonding provide additional reinforcement to the three-dimensional gel network.

Overall, the gel system formed under high-temperature and high-salinity conditions contains abundant carbon-, nitrogen-, and oxygen-containing functional groups. These functional groups contribute to the formation of a stable three-dimensional network through covalent crosslinking, ionic association, and hydrogen-bonding interactions. The synergistic interactions between zwitterionic groups and dissolved salt ions effectively alleviate the charge-screening effect caused by high-salinity brine, which is considered a key mechanism responsible for the excellent salt tolerance of the zwitterionic gel system. Meanwhile, the stable polymer backbone and functional bonds (such as C–N and C–O) maintain structural integrity at elevated temperatures, enabling the gel to maintain reliable performance under harsh reservoir conditions. Notably, different from hydrogen bonding that only occurs between polar groups bearing active hydrogen atoms, the ion-dipole and coordination interactions between salt ions and oxygen-containing functional groups can effectively offset the charge-shielding effect of high-salinity brine on polymer chains, which constitutes an important mechanism for the excellent salt resistance of the zwitterionic gel.

### 2.6. Analysis of Viscoelastic Test Results

The viscoelastic properties of the formed gel were characterized by oscillatory rheological measurements. The storage modulus (G′) and loss modulus (G″) are key rheological parameters used to evaluate the elastic strength, viscous dissipation, and deformation resistance of gel materials. These parameters provide information regarding the structural integrity and mechanical stability of the gel network.

The viscoelastic behavior of the gel system was investigated via oscillatory rheological measurements. Strain sweep measurements were first performed over a strain range of 0.01–100% at 115 °C to determine the linear viscoelastic region (LVR). The results show that the LVR of the gel extends from 0.01% to 10% strain, within which the storage modulus (G′) remains stable with negligible variation. When the strain amplitude exceeds 10%, G′ gradually decreases with increasing strain, marking the transition to the nonlinear viscoelastic regime. Throughout the tested strain range, G′ remained higher than G″, indicating an elasticity-dominated response of the formed gel under the applied deformation conditions. The absence of a G′–G″ crossover in this strain-sweep measurement should not be interpreted as a determination of the rheological gel point, because the measurement was not performed as a time sweep during gel formation.

**Figure 10:**
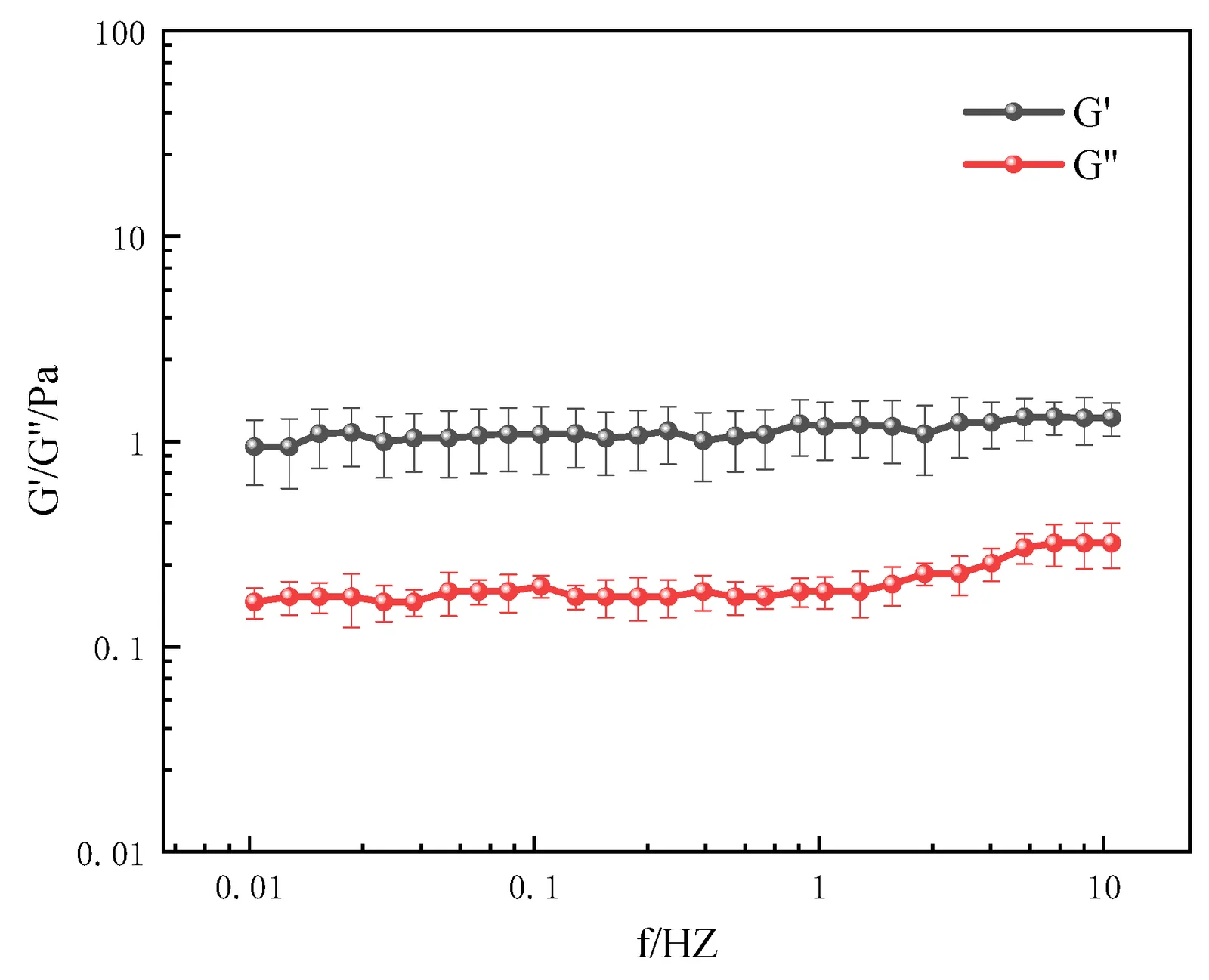
Frequency sweep viscoelastic curve of the zwitterionic polymer gel.

A constant strain of 1%, which is well within the confirmed LVR, was selected for the subsequent frequency-sweep test over a frequency range of 0.1–10 Hz. Rheological measurements were performed in triplicate (n = 3), and the frequency sweep results are presented in Figure 10. Under the test conditions, the average G′ and G″ values of the gel system were 1.115 Pa and 0.205 Pa, respectively. The storage modulus G′ is consistently higher than the loss modulus G″, demonstrating that the material exhibits an elasticity-dominated viscoelastic response under the tested conditions.

Across the tested frequency range, G′ remained relatively stable and consistently higher than G″, indicating that the formed gel exhibited a predominantly elastic viscoelastic response under the selected test conditions. G″ showed a modest increase toward the higher-frequency range but remained substantially lower than G′. These frequency-sweep measurements characterize the viscoelastic behavior of the formed gel and do not provide time-resolved information on the evolution of G′ and G″ during gelation. Therefore, the rheological gel point cannot be determined from the present measurements.

The elastic-dominated and structurally stable viscoelastic properties are consistent with the uniform honeycomb-like porous morphology observed by SEM and the strong intermolecular interactions provided by the zwitterionic and crosslinked structures. More importantly, this stable mechanical behavior enables the gel to withstand water-flow shear and formation pressure within reservoir pores, thereby maintaining structural integrity and providing reliable long-term plugging performance under high-temperature and high-salinity reservoir conditions.

### 2.7. Analysis of Core Flow Experiment Results of Gel System

#### 2.7.1. Analysis of Experimental Results of Single-Tube Plugging Performance

**Figure 11:**
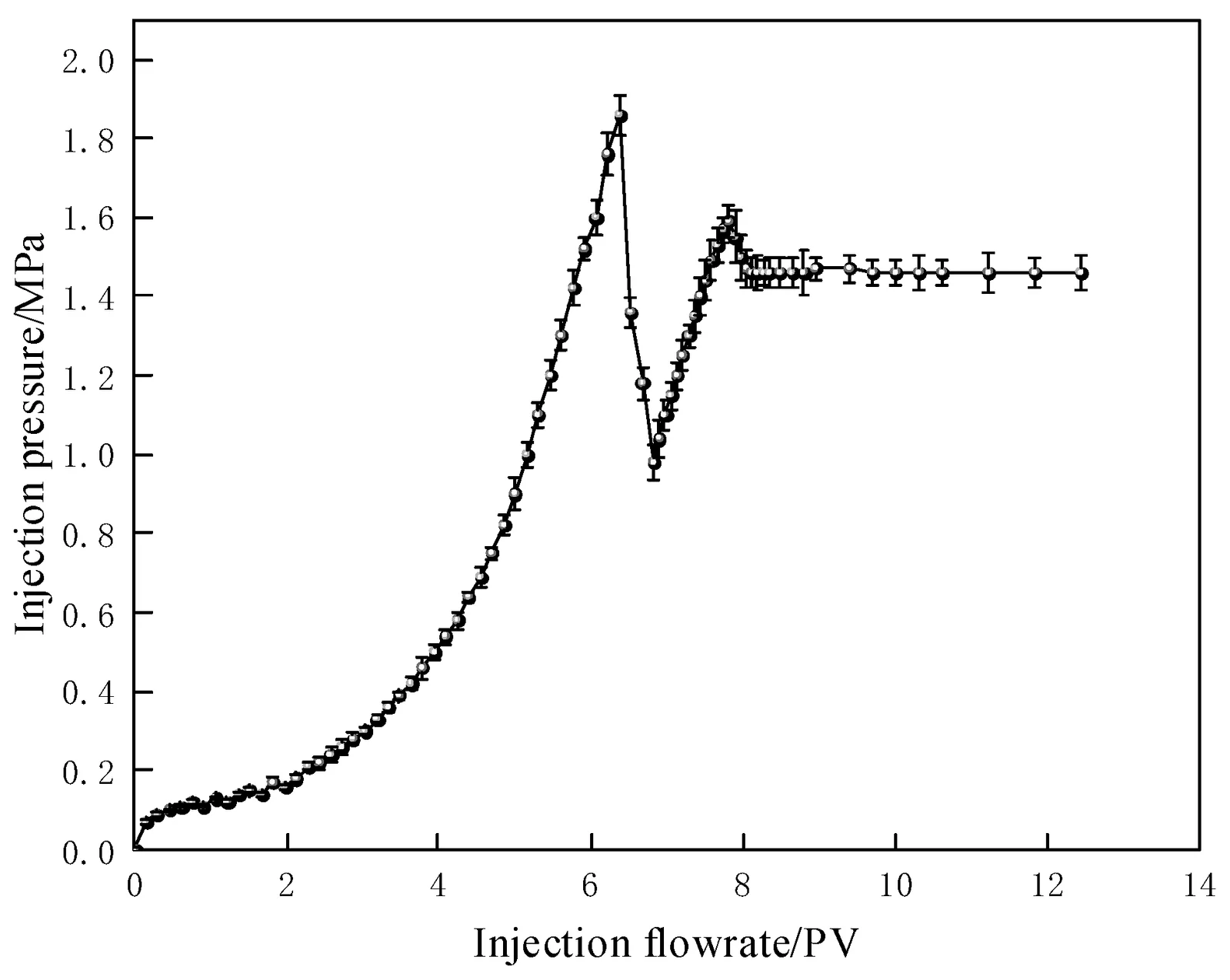
Plugging performance test curve of zwitterionic polymer gel (5.86 mD).

To evaluate the plugging performance of the gel system within reservoir pore structures, core-flooding experiments were conducted under simulated high-temperature and high-salinity conditions representative of the Jianghan Oilfield. Based on commonly applied gel slug sizes in field profile-control operations, 0.2 PV of gel was injected into the core sample. The plugging efficiency, breakthrough pressure, and subsequent pressure response were systematically analyzed to investigate the migration behavior, in situ gelation characteristics, and sealing mechanisms of the gel system. Single-core plugging experiments were performed with three independent core samples (n = 3), and the reported parameters are expressed as mean ± standard deviation. The corresponding experimental results are presented in Figure 11. The core sample used in this experiment exhibited a permeability of 5.86 mD and a porosity of 11.95%.

During the initial injection stage, the core pressure gradually increased with increasing injected gel volume and eventually reached a maximum breakthrough pressure of 1.86 MPa, corresponding to a breakthrough pressure gradient of 18.6 MPa/m. This pressure increase indicates progressive retention, adsorption, and gel accumulation within the pore channels, resulting in effective reduction in flow conductivity and demonstrating the strong initial plugging capability of the gel system.

After reaching the maximum pressure, the core pressure decreased sharply, followed by a rapid increase and subsequent stabilization. This characteristic pressure response suggests that the initially formed plugging structure underwent partial deformation or localized failure under continuous water-flow stress; however, the gel system did not experience complete failure. This behavior can be attributed to its elasticity-dominated viscoelastic properties and dynamic ionic interactions originating from the zwitterionic structure. Under flowing conditions, the gel network can undergo deformation, migration, and structural rearrangement within deeper pore spaces, followed by reformation of secondary plugging structures. These characteristics demonstrate the excellent deformation resistance and secondary plugging capability of the gel system.

After gel treatment, the core permeability decreased from 5.86 mD to 0.87 mD, corresponding to a plugging efficiency of 85.15%. This result confirms that the gel effectively occupied pore spaces and significantly reduced fluid transport capacity within the core. The high plugging efficiency and sustained secondary plugging behavior are attributed not only to the uniform honeycomb-like porous crosslinked structure and stable functional groups identified by SEM and XPS analyses, but also to the elasticity-dominated viscoelastic response revealed by rheological measurements. Overall, the gel system exhibits excellent sealing ability, structural adaptability, and long-term plugging potential, indicating its suitability for conformance control applications in medium-porosity and low-permeability reservoirs under high-temperature and high-salinity conditions.

#### 2.7.2. Analysis of Experimental Results of Double-Tube Shunt

**Figure 12:**
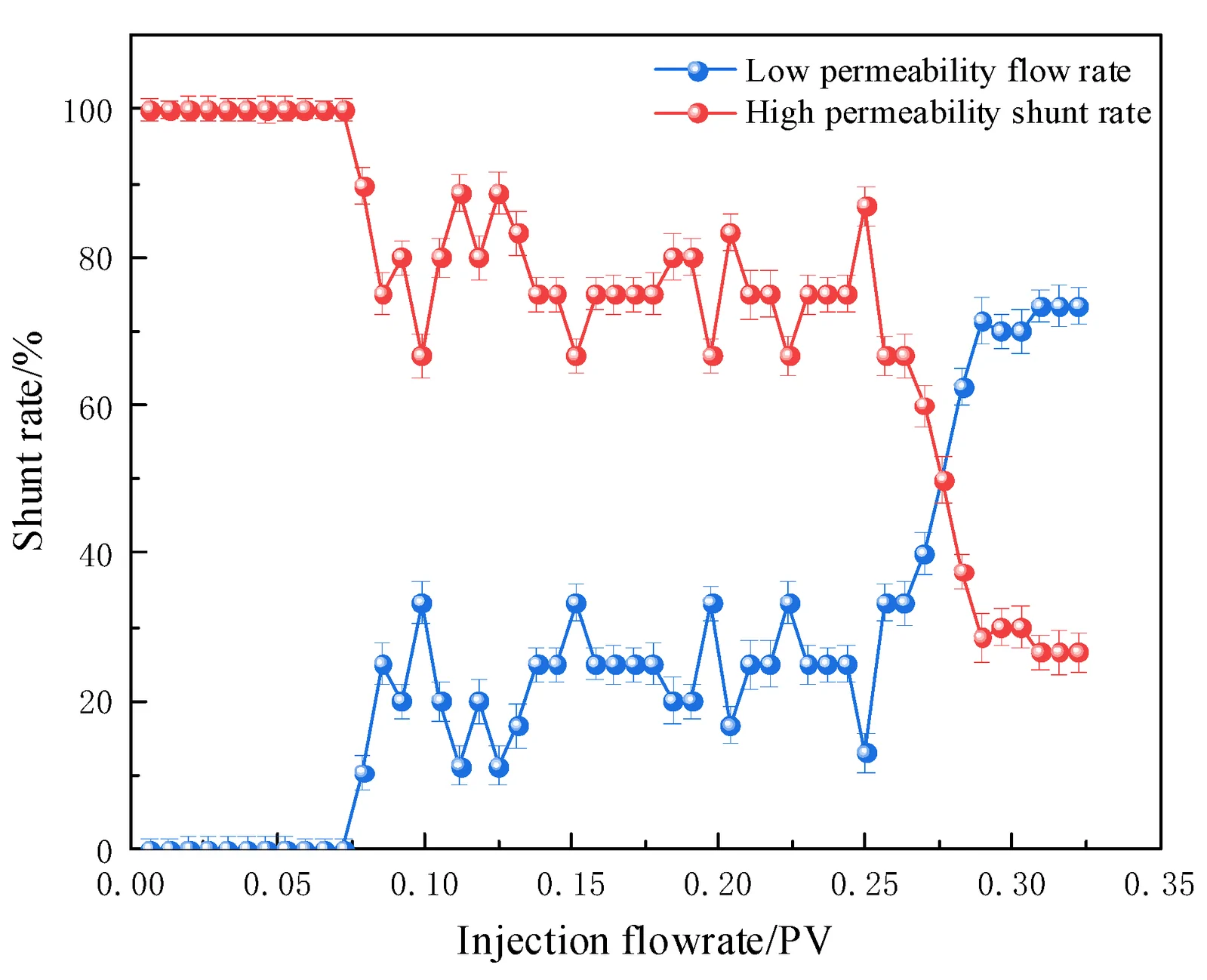
High/low permeability core flow experimental curve of zwitterionic polymer gel (differential 10).

Reservoir heterogeneity often results in preferential water channeling through high-permeability pathways and insufficient sweep of low-permeability zones. To evaluate the profile-modification and flow-diversion capability of the gel system in heterogeneous reservoirs, dual-core flooding experiments were conducted under simulated reservoir conditions of 115 °C and high salinity representative of the Jianghan Oilfield. A total of 0.2 PV of gel was injected, and the injection pressure as well as the diversion ratios between high- and low-permeability cores were continuously monitored to investigate the gel migration behavior, selective plugging performance, and fluid redistribution mechanism. Dual-core diversion experiments were performed with three independent core sets (n = 3), and the reported diversion rates are expressed as mean ± standard deviation. The corresponding experimental results are presented in Figure 12.

The low-permeability core exhibited a permeability of 5.19 mD and a porosity of 10.11%, whereas the high-permeability core showed a permeability of 50.62 mD and a porosity of 20.84%, indicating a significant permeability contrast between the two cores.

During the initial water flooding stage, the diversion ratio in the high-permeability core remained close to 100%, while that in the low-permeability core was nearly zero. This flow distribution behavior reflects the typical characteristics of heterogeneous reservoirs, where injected fluids preferentially migrate through high-permeability channels due to their lower flow resistance.

During the gel injection and plugging stage, the diversion ratio of the high-permeability core decreased rapidly, whereas that of the low-permeability core increased substantially, demonstrating the selective plugging and flow-diversion capability of the gel system. During this process, the gel preferentially entered the high-permeability core, where it was retained and gradually formed a plugging structure within dominant flow channels. The resulting reduction in effective permeability of the high-permeability zone increased the overall injection resistance and promoted fluid redistribution toward the low-permeability core.

The substantial increase in injection pressure observed during gel placement increased the pressure gradient across the dual-core system. According to Darcy’s law, the elevated pressure gradient enhanced the driving force for fluid penetration into the low-permeability core, providing the necessary hydraulic condition for activating previously unswept zones. Meanwhile, the preferential placement and retention of the gel in the high-permeability core reduced its effective permeability through elastic deformation and volumetric filling of flow channels. This mechanism is consistent with the single-core plugging experiment, where the gel achieved an 85.15% reduction in permeability after treatment, confirming its strong sealing capability.

Minor fluctuations in the diversion curves during the gel injection stage were observed, which can be attributed to transient permeability adjustments caused by gel migration, shear deformation, and differences in gelation kinetics. These fluctuations are consistent with the viscoelastic characteristics of the gel system, allowing it to undergo deformation and structural rearrangement under flowing conditions while maintaining overall plugging integrity.

In the subsequent stabilization stage, the diversion ratios gradually reached equilibrium, and the flow contribution of the low-permeability core increased to 73.6%. These results demonstrate that the gel-induced plugging structure possesses strong erosion resistance and effectively achieves selective blocking of high-permeability channels while improving sweep efficiency in low-permeability regions. Therefore, the developed gel system exhibits excellent potential for profile control and enhanced oil recovery applications in heterogeneous reservoirs under high-temperature and high-salinity conditions.

## 3. Conclusions

Considering the challenging reservoir conditions of the Jianghan Oilfield, characterized by medium-porosity and low-permeability formations with a temperature of 115 °C and high salinity, as well as the difficulty of achieving a balance between long-term stability and effective plugging performance in conventional profile-control agents, this study developed and systematically evaluated a phenolic crosslinked zwitterionic polymer gel system. The main conclusions are summarized as follows:

(1) The optimal gel formulation for the target reservoir was determined to consist of 0.3 wt% zwitterionic polymer, 0.03 wt% resorcinol, 0.3 wt% HMTA, and 0.1 wt% thiourea. This formulation exhibited favorable gelation behavior under high-temperature and high-salinity conditions and formed a gel with appropriate mechanical strength for deep-profile control applications.

(2) The developed gel system exhibited favorable long-term thermal and structural stability under the investigated conditions of 115 °C and high salinity. After 140 days of aging at 115 °C in high-salinity (total salinity of 2.486 × 10^5^ mg/L) formation water, the dehydration rate remained below 30%, while SEM observations confirmed the preservation of a uniform honeycomb-like porous crosslinked structure without obvious fracture or collapse. TGA and DSC further characterized the thermal-transition and dehydration behavior of the gel under programmed heating conditions. TGA and DSC indicated that the major thermal event near 171 °C under programmed heating was mainly associated with dehydration rather than severe polymer-backbone degradation. DSC analysis further confirmed that the primary crosslinked network remained stable during the dehydration process, with noticeable network degradation occurring only at higher temperatures. Rheological measurements demonstrated that the gel exhibited an elasticity-dominated viscoelastic response, as evidenced by G′ remaining higher than G″. At the molecular level, the zwitterionic internal ionic associations and oxygen-containing functional groups synergistically contributed to enhanced thermal and salt tolerance, while the dynamic ionic interactions improved resistance to shear deformation and supported secondary plugging behavior.

(3) Core-flooding experiments demonstrated that the gel system achieved a single-core plugging efficiency of 85.15% with a breakthrough pressure of 1.86 MPa, while maintaining excellent secondary plugging capability. In heterogeneous dual-core experiments with an approximately ten-fold permeability contrast, the diversion ratio of the low-permeability core increased to 73.6%, indicating effective flow redistribution and improved sweep efficiency in heterogeneous reservoirs.

(4) The developed zwitterionic polymer gel system exhibited promising laboratory performance under simulated reservoir conditions. Future studies will focus on field pilot trials at representative well groups to further evaluate its deep migration capability, long-term plugging effectiveness, and potential contribution to enhanced oil recovery under actual reservoir conditions.

## 4. Materials and Methods

### 4.1. Experimental Materials and Equipment

The experimental water was collected from produced formation water in Jianghan Oilfield, with a total salinity of 2.5 × 10^4^ mg/L. Prior to use, the water was filtered through filter paper to remove suspended impurities. The water composition is presented in Table 1. In this study, only CaCl_2_-type formation water was used to evaluate the performance of the gel system. Considering the chemical structure of the zwitterionic polymer gel, its behavior may vary in other types of brine environments. For example, Na_2_SO_4_-type brine may weaken the coordination interactions associated with divalent cations, although the shielding effect of Na^+^ ions may still contribute to the anti-polyelectrolyte behavior of the zwitterionic network. In NaHCO_3_-type brine systems, changes in alkalinity may influence the phenolic crosslinking kinetics; however, the corresponding pH range may remain within the effective operating window of the HMTA/resorcinol crosslinking system. A systematic investigation of the gel performance under different brine compositions has not yet been conducted in this work. Future studies will therefore focus on evaluating the effects of various ionic compositions and salinity conditions on gelation behavior, network stability, and plugging performance to further clarify the adaptability of the gel system under diverse reservoir environments.

The chemicals used in this study included 2-acrylamido-2-methylpropanesulfonic acid (AMPS), acrylamide (AM), dimethyldiallylammonium chloride (DMDAAC), ammonium persulfate, sodium bisulfite, sodium hydroxide, hexamethylenetetramine, resorcinol, and thiourea. All reagents were industrial-grade products supplied by Sinopharm Chemical Reagent Co., Ltd, (Shanghai, China).

The core samples used in the experiments were artificial sandstone cores with a diameter of 2.5 cm and a length of 10 cm.

The main experimental instruments included a SU8100 scanning electron microscope (SEM, Tokyo, Japan), a HAAKE MARS 60 rheometer (Karlsruhe, Germany), a DHG-85 electric thermostatic drying oven (Shanghai, China), a UZCQ-50 core flooding system (Nantong, China), a TG-DTA/S thermogravimetric analyzer (TGA, Tokyo, Japan), and a K-Alpha X-ray photoelectron spectrometer (XPS, East Grinstead, UK).

### 4.2. Optimization of Gel System Formulation

#### 4.2.1. Synthesis of Zwitterionic Polymers

The zwitterionic terpolymer was synthesized through free-radical copolymerization in a three-neck flask. First, 80 mL of distilled water was added into the flask, followed by 15.11 g of acrylamide (AM) and 1.89 g of 2-acrylamido-2-methylpropanesulfonic acid (AMPS). The mixture was placed in a water bath maintained at 45 °C and stirred continuously until the monomers were completely dissolved. The pH of the solution was adjusted to approximately 7 using concentrated NaOH solution. After the temperature reached equilibrium, 3 g of dimethyl diallyl ammonium chloride (DMDAAC) was added, and the solution was stirred until homogeneous. Subsequently, nitrogen gas was continuously bubbled through the solution for 30 min to remove dissolved oxygen and minimize oxygen-induced inhibition during polymerization.

**Figure 13:**
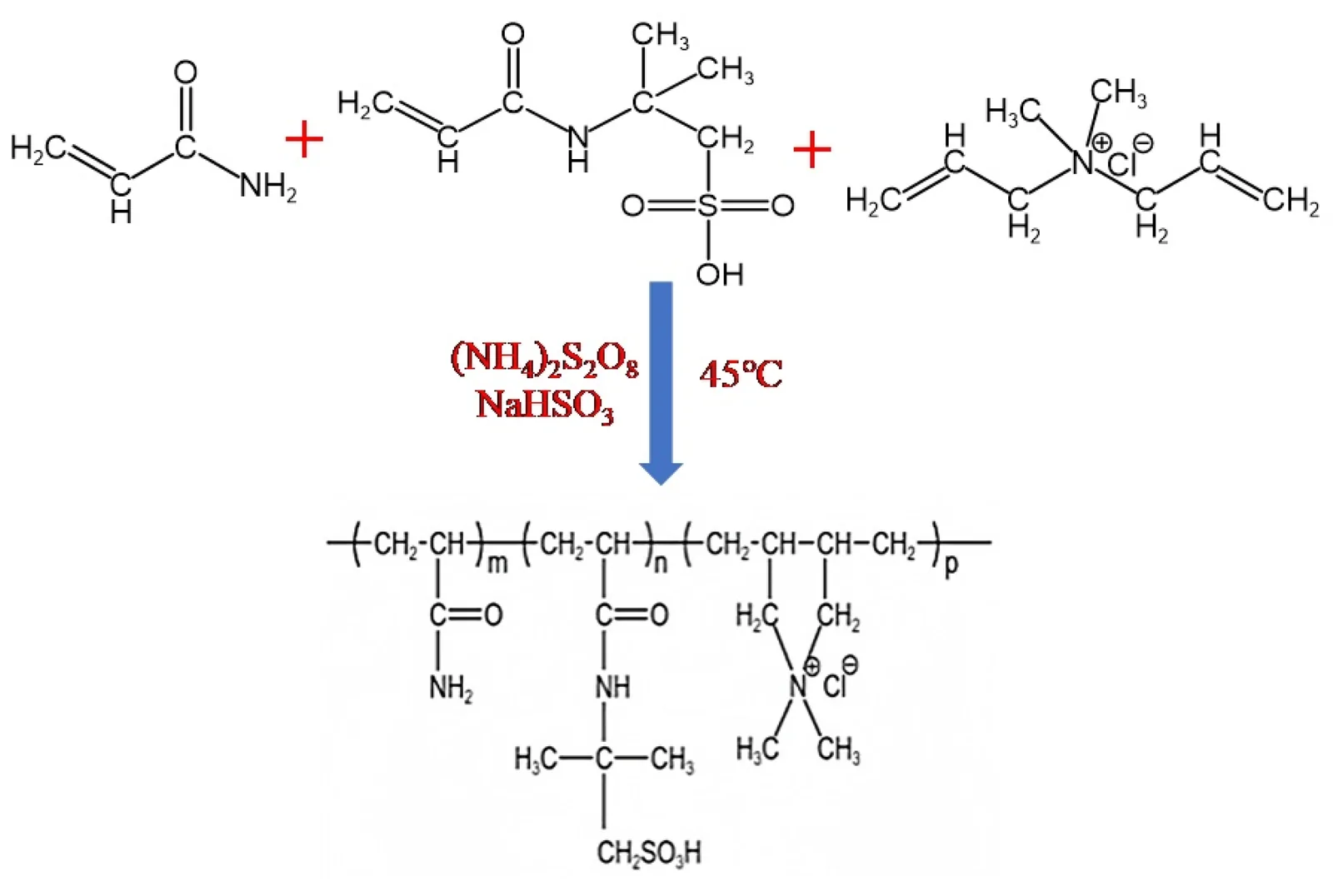
Synthesis process of zwitterionic terpolymer.

Polymerization was initiated by adding 1.4 g of ammonium persulfate ((NH_4_)_2_S_2_O_8_) and 0.6 g of sodium bisulfite (NaHSO_3_). The reaction was allowed to proceed at 45 °C for 20 h. After polymerization, the obtained product was washed three times with deionized water, followed by washing with anhydrous ethanol, and then immersed in ethanol for 24 h for further purification. The purified polymer was dried in a vacuum oven at 45 °C for 48 h. The dried product was subsequently crushed and ground to obtain a white granular terpolymer powder. The AM/AMPS/DMDAAC zwitterionic terpolymer was successfully prepared, as shown in Figure 13. The molecular weight of the synthesized terpolymer was determined by gel permeation chromatography (GPC) using 0.1 mol/L NaNO_3_ aqueous solution as the mobile phase. The measurement was performed at a flow rate of 1.0 mL/min and a column temperature of 30 °C, with narrow-distribution polyethylene oxide standards used for calibration. The weight-average molecular weight (Mw), number-average molecular weight (Mn), and polydispersity index (PDI) were determined to be 3.18 × 10^6^ g/mol, 1.52 × 10^6^ g/mol, and 2.09, respectively.

The synthesized terpolymer integrates three functional monomer units, providing excellent compatibility with formation water, thermal stability, salt tolerance, and gelation capability. The AM units constitute the primary polymer backbone and provide abundant amide groups as reactive sites for subsequent gel crosslinking. The AMPS units introduce sulfonate groups (–SO_3_^−^), which can interact with multivalent cations such as Ca^2+^ and Mg^2+^ through ionic association and coordination interactions, thereby improving salt tolerance and reinforcing the gel network under high-salinity conditions. Meanwhile, the DMDAAC units provide quaternary ammonium groups that interact with sulfonate groups within the polymer chain, forming zwitterionic internal ionic associations. These internal ionic interactions contribute to the anti-polyelectrolyte behavior of the polymer, enabling the gel network to maintain structural stability in highly saline environments and improving its long-term plugging performance. Therefore, this zwitterionic terpolymer was selected as the primary polymer component for subsequent gel formulation and performance evaluations.

#### 4.2.2. Fourier Transform Infrared Spectroscopy Test

**Figure 14:**
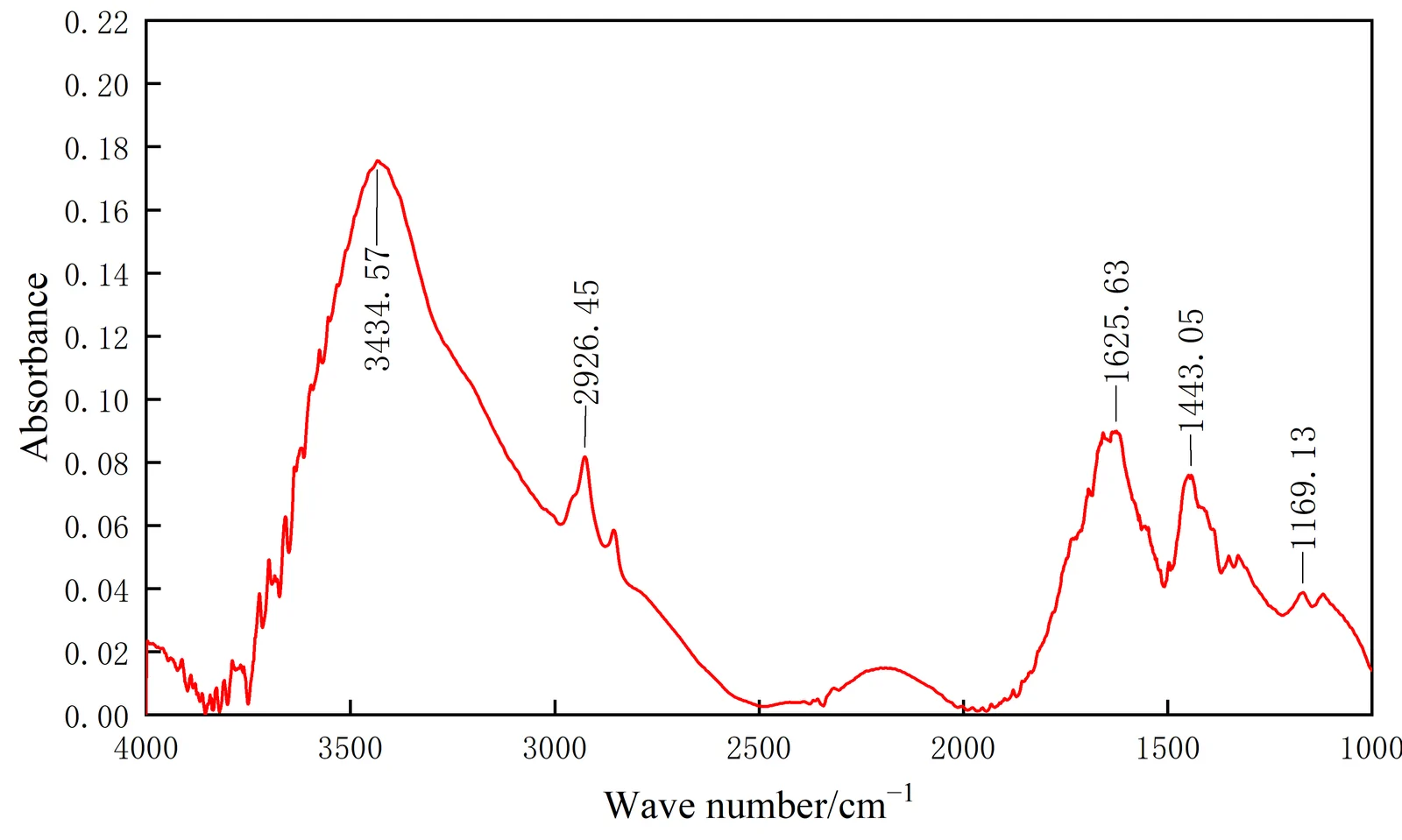
Zwitterionic polymer Fourier transform infrared spectroscopy test results.

The chemical structure of the synthesized zwitterionic terpolymer was characterized by Fourier transform infrared spectroscopy (FTIR), and the corresponding spectrum is shown in Figure 14. A broad and strong absorption band centered at 3434.57 cm^−1^ was observed, which is assigned to the stretching vibration of N–H bonds in amide groups (–CONH_2_) together with the overlapping absorption of O–H stretching vibrations from absorbed water. This characteristic peak confirms the incorporation of acrylamide (AM) units into the polymer backbone. A strong absorption peak at 1625.63 cm^−1^ corresponds to the stretching vibration of the C=O bond in amide groups (amide I band), further verifying the presence of acrylamide structural units. In addition, the absorption peak at 2926.45 cm^−1^ is attributed to the stretching vibration of aliphatic C–H bonds from –CH_2_– and –CH_3_ groups, confirming the formation of a saturated carbon-chain backbone after free-radical polymerization.

A distinct absorption peak at 1169.13 cm^−1^ was observed, which is assigned to the asymmetric stretching vibration of S=O bonds in sulfonate groups (–SO_3_^−^) derived from AMPS units. This characteristic signal provides direct evidence for the successful incorporation of AMPS into the polymer chain. The introduction of sulfonate groups enhances the hydrophilicity of the polymer and provides stable ionic functionalities, which contribute to improved salt tolerance and solution stability under high-salinity conditions. Furthermore, an absorption band near 1443.05 cm^−1^ was mainly associated with the deformation vibration of methyl groups in quaternary ammonium structures and methylene groups within the polymer backbone. This characteristic absorption confirms the successful incorporation of the DMDAAC-derived quaternary ammonium groups into the terpolymer.

The original monomers AM, AMPS, and DMDAAC contain carbon–carbon double bonds (C=C), whose characteristic absorption bands generally appear in the regions of approximately 1600–1680 cm^−1^ and 900–1000 cm^−1^. However, no obvious absorption peaks associated with vinyl groups were observed in the FTIR spectrum of the synthesized polymer. Meanwhile, characteristic absorption signals corresponding to a saturated carbon-chain structure were clearly detected. These results demonstrate that the vinyl groups of the monomers were effectively consumed during polymerization, confirming the successful formation of the polymer backbone through free-radical polymerization.

#### 4.2.3. Analysis of Zwitterionic Polymer XPS Test Results

X-ray photoelectron spectroscopy (XPS) was employed to investigate the elemental composition and chemical bonding states of the synthesized zwitterionic terpolymer. The survey spectrum revealed characteristic photoelectron peaks corresponding to C, N, and O elements, with atomic percentages of 72.96%, 5.53%, and 21.50%, respectively. The dominant carbon content originates from the organic polymer backbone, while the presence of nitrogen and oxygen confirms the successful incorporation of AM-, AMPS-, and DMDAAC-derived functional units into the copolymer chain.

**Figure 15:**
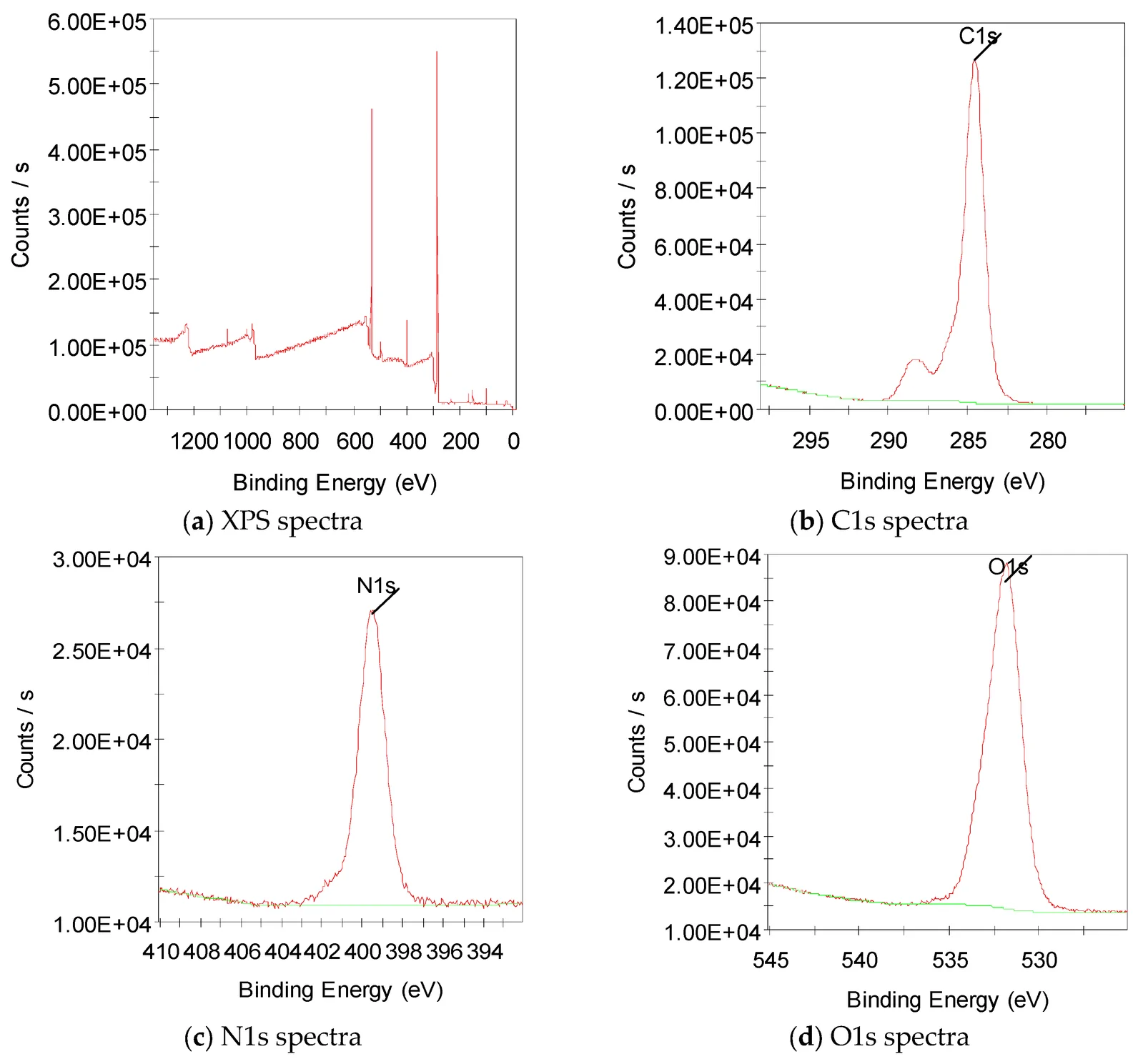
XPS test spectra of zwitterionic polymer.

The high-resolution C 1s spectrum was deconvoluted into three component peaks. The dominant peak located at 284.56 eV was assigned to C–C and C–H bonds associated with the saturated aliphatic backbone of the polymer. The shoulder peak at approximately 286.3 eV was attributed to C–O and C–N bonds, indicating the presence of oxygen- and nitrogen-containing functional groups, including ether/hydroxyl and amide/amine groups. The weak peak centered at approximately 288.5 eV corresponded to the carbonyl carbon (C=O) of amide groups. These C 1s fitting results are consistent with the FTIR analysis (Figure 15), further confirming the successful formation of the AM/AMPS/DMDAAC copolymer structure.

The N 1s spectrum exhibited an asymmetric envelope centered at approximately 399.52 eV and was fitted into two major components. The peak at approximately 399.5 eV was assigned to neutral amide nitrogen (–CONH– and –CONH_2_) originating from AM units. More importantly, a distinct peak at approximately 401.5 eV was attributed to quaternary ammonium nitrogen (–N^+^(CH_3_)_3_) from DMDAAC units. The appearance of this characteristic quaternary ammonium signal confirms the incorporation of cationic DMDAAC-derived groups into the polymer chain. Notably, the binding energy of quaternary ammonium nitrogen (401.5 eV) is slightly lower than that of typical quaternary ammonium salts (approximately 402–403 eV), suggesting that the positive charge is partially influenced by electrostatic interactions with neighboring anionic groups. This binding-energy shift provides evidence for the formation of zwitterionic internal ionic associations within the polymer structure.

The high-resolution O 1s spectrum exhibited a dominant peak at 531.87 eV with an asymmetric tail toward higher binding energy. Peak fitting revealed two contributions. The major component located at approximately 531.9 eV was assigned to oxygen atoms from sulfonate groups (–SO_3_^−^) of AMPS units and carbonyl oxygen (C=O) from amide groups. The shoulder peak at approximately 533.5 eV was attributed to C–O–H and C–O–C species. The presence of sulfonate oxygen signals confirms the successful introduction of AMPS units, where negatively charged sulfonate groups provide ionic interaction sites for quaternary ammonium groups. The slight shift in the O 1s binding energy compared with free sulfonate species further suggests an altered charge environment caused by internal ionic interactions.

Overall, the synthesized zwitterionic terpolymer contains both positively charged quaternary ammonium groups and negatively charged sulfonate groups along the polymer chain. The characteristic binding-energy shifts observed in N 1s and O 1s spectra demonstrate the existence of strong electrostatic interactions between these oppositely charged groups, leading to the formation of zwitterionic internal ionic associations (–N^+^···^−^O_3_S–). This molecular architecture provides the chemical basis for the anti-polyelectrolyte behavior of the polymer and contributes to the enhanced structural stability and salt tolerance of the gel network under high-temperature and high-salinity conditions.

#### 4.2.4. Analysis of Zwitterionic Polymer Zeta Potential Test Results

**Figure 16:**
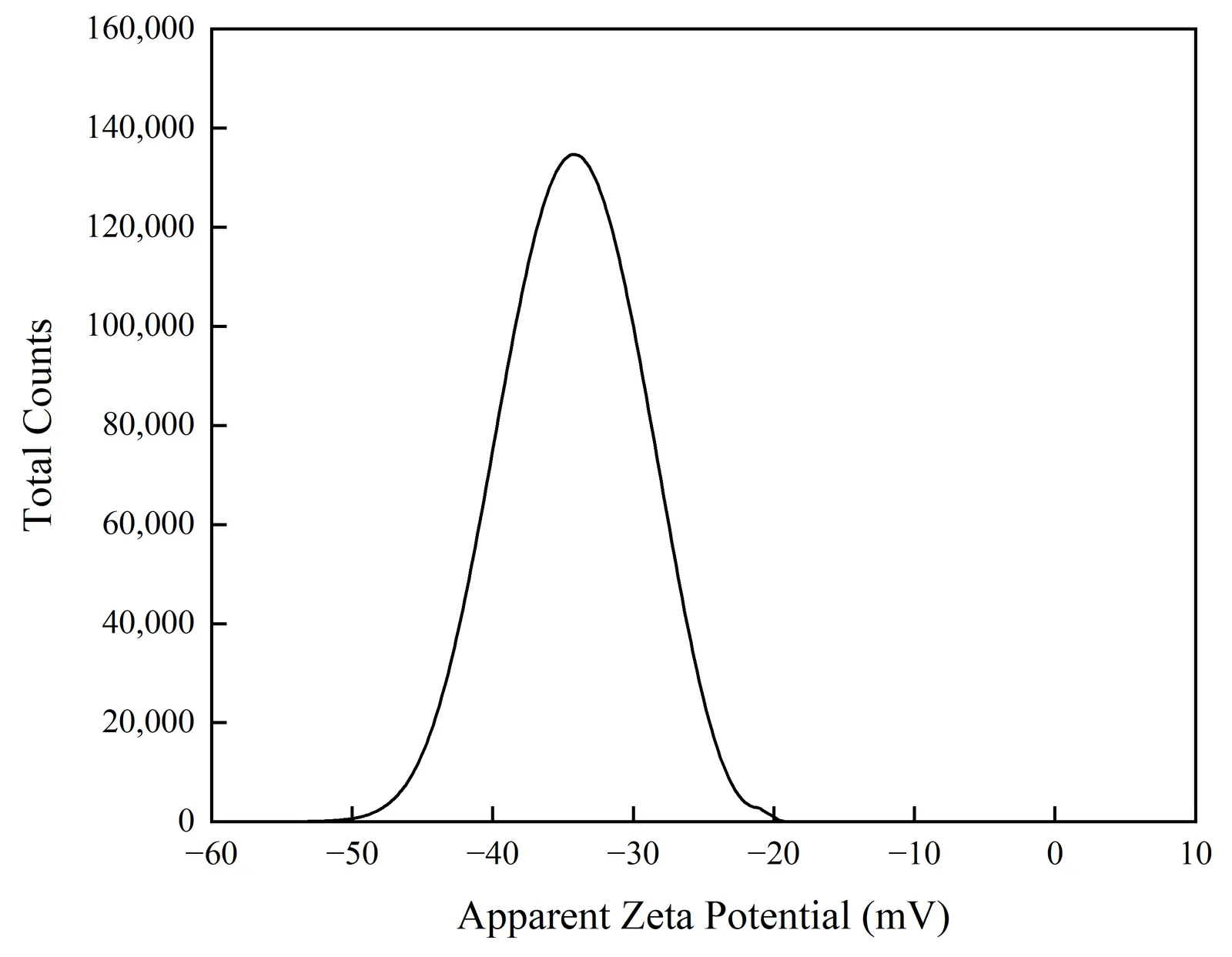
Zeta potential test result curve of zwitterionic polymer.

To further verify the formation of internal ionic associations within the zwitterionic polymer chains and their influence on the charge characteristics of the polymer, zeta potential measurements were conducted to evaluate the apparent surface charge behavior of the polymer solutions. The test results are presented in Figure 16. Under identical measurement conditions (polymer concentration: 0.1 wt%, deionized water, 25 °C), the AM/AMPS copolymer solution exhibited a zeta potential of −48.2 mV, whereas the AM/AMPS/DMDAAC terpolymer solution showed a reduced zeta potential of −34.7 mV with a single-peak distribution.

The AM/AMPS copolymer contains negatively charged sulfonate groups (–SO_3_^−^) derived from AMPS units, resulting in a relatively high negative surface charge. After introducing the cationic DMDAAC units, the terpolymer chain simultaneously contains positively charged quaternary ammonium groups (–N^+^(CH_3_)_3_) and negatively charged sulfonate groups. In aqueous media, these oppositely charged groups can approach each other through electrostatic interactions and form intramolecular ionic associations. Consequently, part of the negative charge is compensated by the neighboring quaternary ammonium groups, reducing the apparent charge density of the polymer chain and leading to a decrease in the absolute zeta potential value from −48.2 mV to −34.7 mV.

The reduced zeta potential of the AM/AMPS/DMDAAC terpolymer provides further evidence for the formation of zwitterionic internal ionic associations, which is consistent with the XPS results. These internal ionic interactions are expected to contribute to the unique salt-responsive behavior and structural stability of the subsequent gel system under high-salinity reservoir conditions.

#### 4.2.5. Gel Preparation

**Figure 17:**
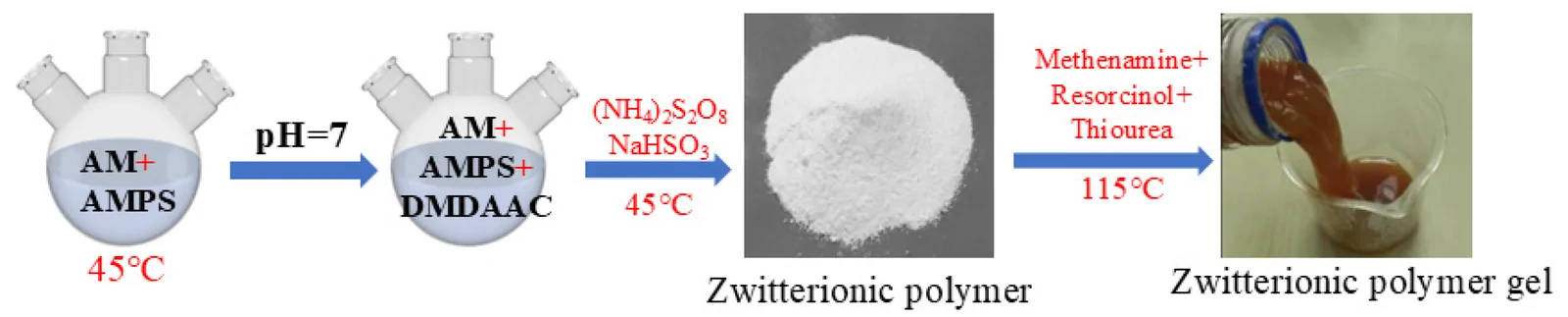
Preparation flow chart of zwitterionic polymer gel.

A total of 200 mL of treated formation water was transferred into a 250 mL beaker and preheated to 70 °C in a thermostatic water bath. The zwitterionic polymer powder was gradually added under continuous mechanical stirring at 600 rpm and mixed for 1 h until a homogeneous polymer solution was obtained. Subsequently, the phenolic crosslinking agents and thermal stabilizer were added sequentially, followed by thorough mixing to ensure uniform dispersion. The prepared gel precursor solution was then transferred into sealed glass bottles and aged in an oven at 115 °C to initiate gelation. The gelation process was monitored at 1 h intervals, and the gel appearance and gelation state were systematically recorded throughout the aging period. The preparation process of zwitterionic polymer gel is shown in Figure 17.

### 4.3. Experimental Method of Performance Evaluation

A predetermined amount of gel sample was weighed and placed into a sealed reactor, and the initial mass (m_1_) was recorded. The reactor was then transferred into a high-temperature oven for thermal aging. During the aging process, the dehydration behavior of the gel was monitored at predetermined time intervals. At each aging time, the gel sample was removed, cooled to room temperature, and weighed to obtain the dehydrated mass (m_2_). The dehydration rate (T) was calculated according to Equation (1):

(1) $$T=\frac{m_{1}-m_{2}}{m_{1}}\times 100\%$$

Thermogravimetric analysis (TGA): The thermal stability of the gel was evaluated using a thermogravimetric analyzer (Rigaku, Tokyo, Japan). Before measurement, gel samples were purified and dried. The TGA experiments were performed under a nitrogen atmosphere from room temperature to 300 °C at a heating rate of 5 °C/min. The thermal stability and degradation characteristics of the gel were evaluated based on the corresponding weight-loss curves.

Differential scanning calorimetry (DSC): DSC was performed to investigate the thermal transition behavior and thermal stability of the gel. Prior to measurement, fully gelled samples were freeze-dried, and approximately 5–10 mg of dried gel powder was sealed in an aluminum crucible. An empty aluminum crucible was used as the reference. The measurements were conducted under a nitrogen atmosphere with a flow rate of 50 mL/min. The temperature was increased from room temperature to 300 °C at a heating rate of 5 °C/min, identical to the TGA conditions to ensure comparability between the two analyses. The heat flow variation as a function of temperature was recorded to analyze water removal, structural transitions, and thermal decomposition behavior of the gel.

Scanning electron microscopy (SEM) analysis: The microstructure and surface morphology of the gel were characterized using scanning electron microscopy (SEM). Before observation, the gel samples were freeze-dried, cryogenically fractured, and sputter-coated with gold. The prepared samples were subsequently mounted onto the SEM stage for imaging. SEM images obtained at different magnifications were analyzed to evaluate the pore structure, surface morphology, and three-dimensional network characteristics of the gel.

X-ray photoelectron spectroscopy (XPS) test: The surface chemical composition and bonding states of the gel were analyzed using X-ray photoelectron spectroscopy (XPS). Prior to measurement, freeze-dried gel samples were pressed into thin pellets and transferred into the XPS chamber. The binding energies were calibrated using the C 1s peak at 284.8 eV as the reference. Survey spectra were collected to determine the elemental composition of the gel surface, while high-resolution spectra were fitted to identify the corresponding chemical bonding environments and functional groups. The obtained results were used to investigate the surface chemical structure and ionic interaction characteristics of the gel network.

Rheological test: The viscoelastic properties of the gel were measured using a HAAKE MARS 60 rotational rheometer equipped with a 20 mm parallel-plate geometry. To minimize wall-slip effects caused by the low modulus of the soft gel, 800-mesh abrasive paper was attached to both plate surfaces to enhance interfacial friction. The measurement gap was set to 1000 μm to maintain the integrity of the three-dimensional porous gel structure and avoid mechanical compression damage. Strain sweep measurements were initially performed at 115 °C over a strain range of 0.01–100% to determine the linear viscoelastic region (LVR). The LVR was identified to extend to approximately 10% strain, confirming that the subsequent frequency sweep tests conducted at 1% strain were within the linear regime. Frequency sweep measurements were then carried out at a constant strain of 1% over a frequency range of 0.1–10 Hz. The storage modulus (G′) and loss modulus (G″) at 1 Hz were recorded to evaluate the mechanical strength, viscoelastic behavior, and structural stability of the gel system. Zero-gap calibration and baseline correction were performed before each measurement to minimize systematic errors.

### 4.4. Core Flow Experiment of Gel System

#### 4.4.1. Evaluation of Single-Tube Plugging Performance

(1) The artificial cores were dried to constant weight and subsequently placed in a vacuum saturation apparatus. The cores were evacuated for 2 h, after which formation water was introduced while maintaining vacuum conditions for an additional 24 h. The saturated cores were then maintained at atmospheric pressure for 12 h to ensure complete saturation.

(2) Based on the field parameters of the target reservoir block, the field injection rate was calculated to be 0.16–0.38 PV/a according to Equation (2):

(2) $$V=\frac{180P_{max}N_{min}}{\left(L^{2}\emptyset \right)}$$

where V represents the field injection rate (PV/a), N_min_ is the minimum apparent water absorption index of the reservoir layer (m^3^/(d·m·MPa)), P_max_ is the maximum wellhead injection pressure (MPa), L is the injection–production well spacing (m), and φ represents reservoir porosity (%).

The equivalent laboratory injection rate was subsequently determined using the field-to-laboratory conversion relationship (Equation (3)):

(3) $$v_{lab}=\frac{Vh\emptyset \pi d^{2}}{20,957.6}$$

where v_lab_ is the laboratory core flooding rate (mL/min), h is the effective reservoir thickness (m), and d is the core diameter (cm). Based on this calculation, the final laboratory injection rate was selected as 0.5 mL/min.

(3) The fully saturated cores were mounted in a core holder, and the confining pressure was maintained at 2 MPa higher than the displacement pressure. The core holder was then heated to 115 °C to simulate reservoir conditions. Formation water was injected at a constant rate of 0.5 mL/min until a stable pressure response was achieved. The stabilized pressure was recorded, and the initial water permeability (K_w_) was calculated.

(4) The gel precursor solution prepared with formation water was transferred into an intermediate container. A constant back pressure was applied at the outlet of the core holder, and the gel solution was injected into the core in the reverse direction at 0.5 mL/min to improve placement uniformity and minimize premature preferential flow. After injection of the designed pore volume (PV), the injection valve was closed. The core system was maintained at 115 °C for 24 h to allow sufficient gel maturation and the formation of a stable plugging structure.

(5) After gel aging, forward water flooding was conducted at a constant rate of 0.5 mL/min. The breakthrough pressure and stabilized displacement pressure were recorded, and the post-treatment water permeability (K_w′_) was calculated. The plugging efficiency (η) was determined according to Equation (4):

(4) $$\eta =\frac{\left(K_{w}-K_{w^{\prime }}\right)}{K_{w}}\times 100$$

where η represents the plugging efficiency (%), Kw is the initial water permeability (mD), and Kw′ is the water permeability after gel treatment (mD).

**Figure 18:**
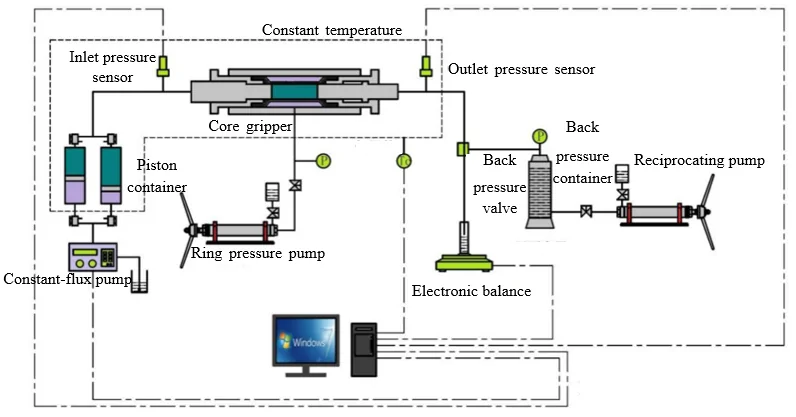
Core flow experimental device diagram.

In addition, the flow diversion behavior between high- and low-permeability cores was evaluated using a dual-core flooding system. The diversion ratio of each core was continuously monitored during water flooding and gel treatment to investigate the selective plugging capability and flow redistribution behavior of the gel system. The experimental setup and procedures are illustrated in Figure 18.

#### 4.4.2. Double-Tube Shunt Experiment

(1) Initial water flooding was first performed on the dual-core system at a constant injection rate of 0.5 mL/min. The inlet pressure and flow rate of each core were continuously monitored and recorded at 1 min intervals. The flooding process was terminated after the pressure and flow distribution reached a stable state.

(2) The prepared gel precursor solution (gelant) was then injected into the dual-core system at a constant rate of 0.5 mL/min. During gelant injection, the inlet pressure was recorded at 1 min intervals. Injection was stopped after 0.1 PV of gelant was introduced into the core system. Subsequently, the inlet and outlet valves were closed, and the core system was maintained at 115 °C for 24 h to allow gel maturation and the establishment of a stable plugging structure.

(3) After thermal aging, the inlet and outlet valves were reopened, and post-gel water flooding was conducted under the same conditions as the initial flooding stage. The pressure response and flow distribution between the high- and low-permeability cores were recorded after reaching a stable condition.

(4) The flow diversion capability of the gel system was evaluated by comparing the flow distribution between the high- and low-permeability cores before and after gel treatment. The diversion ratio was calculated according to Equation (5):

(5) $$Shuntrate=\frac{Q_{1}}{Q_{2}}\times 100\%$$

where Shuntrate represents the diversion ratio of an individual core (%), Q_1_ is the flow rate through the corresponding core (mL/min), and Q_2_ is the total flow rate of the dual-core system (mL/min).

## Figures and Tables

**Table 1:** Analysis Data Sheet of Formation Water.

Ion Mass Concentration/mg·L^−1^						Total Mineralization/mg·L^−1^	Water Classifying
Cl^−^	Na^+^+K^+^	HCO_3_^−^	Ca^2+^	Mg^2+^	SO_4_^2−^		
145,960	85,340	570	12,280	3210	1240	248,600	CaCl_2_

## Data Availability

The original contributions presented in this study are included in the article. Further inquiries can be directed to the corresponding author(s).

## Nomenclature

Abbreviations	
AM	Acrylamide
AMPS	2-acrylamide-2-methylpropanesulfonic acid
DMDAAC	Dimethyldiallylammonium chloride
TGA	Thermogravimetric analysis
DTG	Derivative thermogravimetry
SEM	Scanning electron microscopy
XPS	X-ray photoelectron spectroscopy
BE	Binding energy
G′	Storage modulus
G″	Loss modulus
LVR	Linear viscoelastic region
PV	Pore volume
Physical Symbols	
T	Dehydration rate of gel
m_1_	Initial mass of gel before thermal aging
m_2_	Gel mass after dehydration at a given aging time
V	Field injection rate
P_max_	Maximum wellhead injection pressure
N_min_	Minimum apparent water absorption index of the oil layer
L	Injection-production well spacing
Φ	Reservoir porosity
v_lab_	Laboratory core injection rate
h	Effective thickness of the target reservoir
d	Core diameter
η	Plugging efficiency
K_w_	Initial water-phase permeability
K_w_′	Water-phase permeability after gel plugging
Q_1_	Flow rate of a single core sample
Q_2_	Total flow rate of the dual-core system
Units	
wt%	Mass fraction
°C	Degree Celsius
mg/L	Milligram per liter
mD	Millidarcy
MPa	Megapascal
MPa/m	Megapascal per meter
Pa	Pascal
mL/min	Milliliter per minute
cm	Centimeter
mm	Millimeter
μm	Micrometer
kV	Kilovolt
eV	Electron volt
rpm	Revolutions per minute
h	Hour
d	Day
a	Year

## References

[1] Li F., Luo Y., Luo X., Wang L., Nagre R.D. (2016). Experimental study on a new plugging agent during CO_2_ flooding for hetero-geneous oil reservoirs: A case study of Block G89-1 of Shengli oilfield. J. Pet. Sci. Eng..

[2] Mi L., Jiang H., Pei Y., Li J., Tian J., Xin Y., Yan B., Killough J.E. (2016). Microscopic oil and water percolation characteristic inves-tigation of water flood reservoir in ultrahigh water cut period. SPE Trinidad and Tobago Section Energy Resources Conference.

[3] Wang D., Tand J. (1986). Production Technology of Daqing Oil-Field During its High Water-Cut Stage. SPE International Oil and Gas Conference and Exhibition in China.

[4] Zou X., Yu G., Huang Y., Thanh T., Francisco C., Li Y., Tian X., Tong J., Ge L., Halomoan P.M. (2013). Best Practices and Application of Integrated Fit for Purpose Technologies to Revitalise High Water Cut Mature Fields—A Case History from Offshore South China. IPTC 2013: International Petroleum Technology Conference.

[5] Zhao Y., Jiang H., Li J., Wang C., Gao Y., Yu F., Su H. (2017). Study on the classification and formation mechanism of microscopic remaining oil in high water cut stage based on machine learning. Abu Dhabi International Petroleum Exhibition and Conference.

[6] Hakiki F., Maharsi D.A., Marhaendrajana T. (2015). Surfactant-Polymer Coreflood Simulation and Uncertainty Analysis Derived from Laboratory Study. J. Eng. Technol. Sci..

[7] Zhang L., Pu C., Sang H., Zhao Q. (2015). Mechanism study of the cross-linking reaction of hydrolyzed polyacrylamide/Ac3Cr in formation water. Energy Fuels.

[8] Zhang L., Zheng L., Pu J., Pu C., Cuit S. (2016). Influence of Hydrolyzed Polyacrylamide (HPAM) Molecular Weight on the Cross-Linking Reaction of the HPAM/Cr^3+^ System and Transportation of the HPAM/Cr^3+^ System in Microfractures. Energy Fuels.

[9] Zhang L., Xu H., Pu C., Yuan G., Leng G., Zhang C. (2017). An experimental study on positioning conformance control of starch gelant in the heterogeneous porous media by using CT technology. J. Ind. Eng. Chem..

[10] Zhang L., Pu C., Cui S., Nasir K., Liu Y. (2017). Experimental Study on a New Type of Water Shutoff Agent Used in Fractured Low Permeability Reservoir. J. Energy Resour. Technol.-Trans. ASME.

[11] Zhu D., Bai B., Hou J. (2017). Polymer Gel Systems for Water Management in High-Temperature Petroleum Reservoirs: A Chemi-cal Review. Energy Fuels.

[12] Sharma P., Kudapa V.K. (2022). Study on the effect of cross-linked gel polymer on water shutoff in oil wellbores. Mater. Today Proc..

[13] Leng J., Wei M., Bai B. (2021). Review of transport mechanisms and numerical simulation studies of preformed particle gel for con-formance control. J. Pet. Sci. Eng..

[14] Shen A., Hu A., Qiao Z., Zheng J., She M., Pan L. (2024). Development and preservation mechanism of deep and ultra-deep car-bonate reservoirs. Sci. China-Earth Sci..

[15] Zhang K., Liu X.-F., Wang D.-B., Zheng B., Chen T.-H., Wang Q., Bai H., Yao E.-D., Zhou F.-J. (2024). A review of reservoir damage during hydraulic fracturing of deep and ultra-deep reservoirs. Pet. Sci..

[16] Bai Y., Xiong C., Wei F., Li J., Shu Y., Liu D. (2015). Gelation Study on a Hydrophobically Associating Polymer/Polyethylenimine Gel System for Water Shut-off Treatment. Energy Fuels.

[17] Vargas-Vasquez S.M., Romero-Zeron L.B. (2008). A review of the partly hydrolyzed polyacrylamide Cr(III) acetate polymer gels. Pet. Sci. Technol..

[18] Song H., Pope G.A., Mohanty K.K. Thermal Stability of Acrylamide-Based Polymers at High Temperature and High Salinity. Proceedings of the SPE Canada Heavy Oil Conference.

[19] Wang J., AlSofi A.M., AlBoqmi A.M. (2016). Development and evaluation of gel-based conformance control for a high salinity and high temperature carbonate. SPE EOR Conference at Oil and Gas West Asia.

[20] Liu J., Zhong L., Wang C., Li S., Yuan X., Liu Y., Meng X., Zou J., Wang Q. (2021). Investigation of a high temperature gel system for application in saline oil and gas reservoirs for profile modification. J. Pet. Sci. Eng..

[21] Duran-Valencia C., Bai B., Reyes H., Fajardo-Lopez R., Barragan-Aroche F., Lopez-Ramirez S. (2014). Development of enhanced nanocomposite preformed particle gels for conformance control in high-temperature and high-salinity oil reservoirs. Polym. J..

[22] Amir Z., Said I.M., Jan B.M. (2019). In situ organically cross-linked polymer gel for high-temperature reservoir conformance control: A review. Polym. Adv. Technol..

[23] Parmar P., Khanikar B., Saha R., Jeeru L., Agarwal A., Bera A. (2025). Smart Crosslinked Polymer Gels for Water Shutoff in Oil Reservoir: A Comprehensive Review on Materials, Mechanisms, and Field Applications. Arab J. Sci. Eng..

[24] Li C., Li Z., Zhou M., Tu H. (2024). Synthesis and Properties of Temperature- and Salt-Resistant CP(AM-AMPS-DMDAAC) Nano-spheres for Oil Displacement. ACS Appl. Polym. Mater..

[25] Gao S., Lin D., Li A., Deng L., Dong A., Zhang J. (2025). A zwitterionic polymer filtrate reducer with enhanced anti-polyelectrolyte effect for high-temperature and high-salinity conditions. Chem. Eng. J..

[26] Liu Y., Zhou L., Tang Y., Liu Q., Li W., Zhang Y. (2023). Synthesis and Characterization of a Res-in/Acrylamide-2-acrylamide-2-methylpropane Sulfonate-Diallyl Dimethyl Ammonium Chloride N vinyl-2-pyrrolidinone Polymer Microcapsule Gelling Agent for Oil and Gas Field Transformation. ACS Omega.

[27] Li X., Fu M., Hu J. (2024). Preparation and Performance Evaluation of Temperature-Resistant and Salt-Resistant Gels. Gels.

[28] Li X., Liu S., Zhang J., Han S., Zhao L., Xu A., Wang J., Zhou F., Li M. (2024). High-Temperature-Resistant Profile Control Sys-tem Formed by Hydrolyzed Polyacrylamide and Water-Soluble Phenol-Formaldehyde Resin. Gels.

[29] Guo H., Ge J., Zhao S., Xu Y., Zhou D., Tao Z. (2022). Performance Evaluation of High-Strength Polyethyleneimine Gels and Syn-eresis Mechanism under High-Temperature and High-Salinity Conditions. SPE J..

[30] He Y., Alamri H., Kawelah M., Gizzatov A., Alghamdi M.F., Swager T.M., Zhu S.S. (2020). Brine-Soluble Zwitterionic Copolymers with Tunable Adsorption on Rocks. ACS Appl. Mater. Interfaces.

[31] Su Y., Wu T., Zheng L., Yang W.-G., Liu T., Sun H., Zhou S., Wang S., Yang D. (2025). Electrostatically Enhanced Swelling Stabil-ity and Mechanics of Polyzwitterionic P(AM/AMPS/DMC) Hydrogels for High-Temperature and High-Salinity Reservoir En-vironment. Energy Fuels.

